# Escitalopram promotes recovery from hand paresis in cortical sensori-motor stroke: a randomized, double-blind, placebo-controlled longitudinal study

**DOI:** 10.1186/s12984-026-01888-w

**Published:** 2026-01-31

**Authors:** Vanessa Vallesi, Werner Krammer, Andrea Federspiel, John H. Missimer, Manuela Pastore-Wapp, Georg Kägi, Roland Wiest, Bruno J. Weder

**Affiliations:** 1 Support Centre for Advanced Neuroimaging (SCAN), Institute for Diagnostic and Interventional Neuroradiology, Inselspital, Bern University Hospital, University of Bern, Bern, Switzerland; 2 Advanced Imaging Research (AIR) Group, Swiss Paraplegic Research, Nottwil, Switzerland; 3 Department of Neurology, Kantonsspital St.Gallen, St.Gallen, Switzerland; 4 Laboratory of Biomolecular Research, Paul Scherrer Institute, Villigen, Switzerland; 5 Gerontechnology & Rehabilitation Group, ARTORG Center for Biomedical Engineering Research, University of Bern, Bern, Switzerland; 6 Department of Neurology, Bern University Hospital, Inselspital, University of Bern, Bern, Switzerland

**Keywords:** Ischemic stroke, Escitalopram treatment, Hand motor recovery, fMRI, Imitation task, Neuronal reorganization

## Abstract

**Background:**

Selective serotonin reuptake inhibitors (SSRIs) have been proposed to support post-stroke motor recovery, but evidence for domain-specific behavioral effects and associated neural mechanisms remains limited. This study examined whether early escitalopram administration influences recovery of within-hand motor dexterity following first-ever sensorimotor stroke affecting the pre- or postcentral gyrus.

**Methods:**

In a double-blind, placebo-controlled trial, participants were randomized to receive escitalopram or placebo during the first three months post-stroke. Motor dexterity was assessed behaviorally and with fMRI using an imitation-based task requiring observation and execution of grasp–regrasp movements. Measurements were acquired at baseline, three months, and nine months. Behavioral effects were analyzed using nonparametric statistics and a complementary permutation framework to assess robustness under small-sample conditions.

**Results:**

The escitalopram group showed greater improvement in Jebsen–Taylor Test subtest 1 from baseline to three months and in finger gaiting from three to nine months, with both effects supported by permutation testing. fMRI revealed increased activation in a left-hemispheric premotor–opercular–striatal network (OP6, BA44, anterior insula, posterior putamen) and in right premotor subarea 6v3 during motor execution in the escitalopram group. The placebo group, by contrast, exhibited increased activity in the left mediodorsal thalamus at nine months, consistent with compensatory recruitment.

**Conclusion:**

Although based on a small, highly specific cohort, the findings suggest that early escitalopram administration may facilitate recovery of fine motor control by supporting normalization of task-relevant cortical and subcortical networks. The placebo group’s delayed recovery pattern, characterized by thalamic overactivation, is compatible with compensatory executive engagement. Larger studies are needed to confirm these preliminary but mechanistically informative results.

**Supplementary Information:**

The online version contains supplementary material available at 10.1186/s12984-026-01888-w.

## Introduction

The assessment of activity imitation is a central aspect of the World Health Organization’s (WHO) International Classification of Functioning, Disability, and Health (ICF) [1]. Up to 85% of stroke patients suffer from sensorimotor hemiparesis in the acute stage of ischemic stroke, and between 55 and 75% retain varying degrees of hemiparesis beyond 6 months post-stroke [2–4] Loss of skilled hand function in hemiparesis is mediated through a critical impairment of the sensorimotor network, which disrupts the ability to produce independent and coordinated finger movements [5, 6]. Diminished manual dexterity and somatosensory function contribute significantly to differences in functional capacity. However, these factors are often overlooked during routine clinical examinations, which do not include examinations of specific hand tasks [7–9].

Behavioral studies have shown that most motor recovery occurs within the first four weeks to three months post-stroke and then gradually tapers off [10]. Thus, this early phase might represent a critical time window for cortical neuroplasticity that can be used to administer interventional medical treatment.

Selective serotonin reuptake inhibitors (SSRIs) constitute an important class of medications. They are neuromodulatory agents that act pre- and postsynaptically, influencing excitatory glutamatergic and inhibitory gamma-aminobutyric acid (GABA) transmission [11]. Paradoxically, although glutamate-mediated hyperexcitability can aggravate tissue damage, shifts in the excitatory/inhibitory balance after the hyperacute phase might actually facilitate plasticity [12–14]. This may occur through the emergence of novel cortical rhythms, more flexible stimulus-response relationships in single neurons, or, most importantly, through long-term potentiation (LTP) of perilesional synapses [15–17]. LTP is the long-lasting enhancement of synaptic neurotransmission, resulting from the synchronous activity of pre- and post-synaptic elements. It is a key mechanism for memory and learning in the cortex [18–20]. Although initial reports were inconclusive, both animal and human studies now provide evidence of motor recovery after stroke with SSRIs. Experimental studies in animals showed that citalopram promotes sensorimotor recovery within days after stroke, presumably by enhancing neurogenesis [21–23]. A short delay in initiating treatment might have been of importance according to Chen et al. [21]. From a clinical perspective, a Cochrane review concluded that SSRIs may improve outcomes such as dependence, disability, neurological impairment, anxiety, and depression following stroke [24]. However, a subsequent study was inconclusive [25]. More recently, findings from a major systematic review and meta-analysis indicated that SSRIs are effective in preventing and treating depression, as well as improving anxiety, motor function, cognitive function, and dependence in post-stroke patients, whereby citalopram and escitalopram were not distinguished [26]. Similar findings were reported by Su et al. [27]. In healthy volunteers, Molloy et al. [28] observed signs of adaptive sequence motor learning in fMRI scans after seven days of escitalopram intake, coinciding with steady-state plasma levels.

**Fig. 1:**
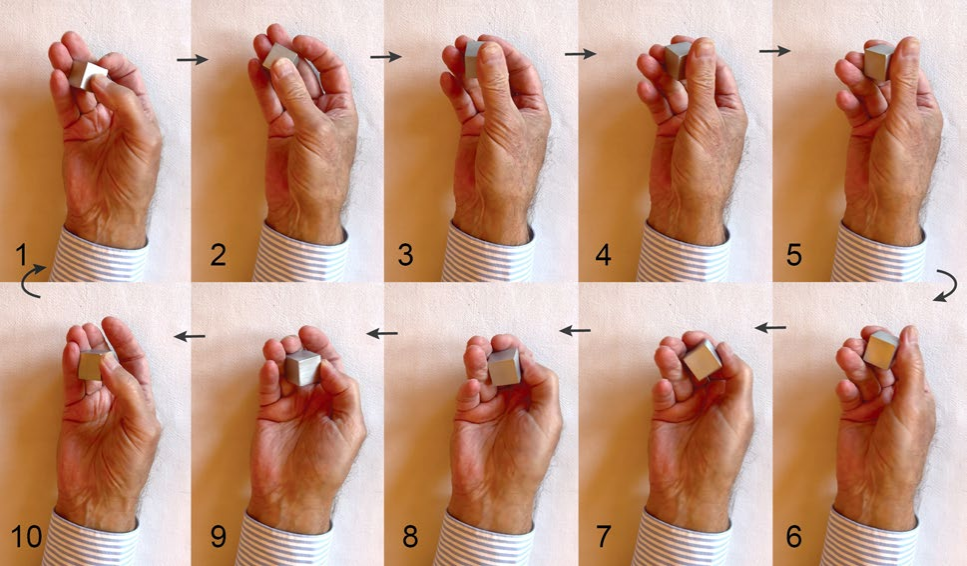
Single grasp–regrasp cycle. Image sequence (frames 1–10) illustrating one grasp–regrasp cycle performed during the fMRI task at 1 Hz. The movement is purely prehensile: the thumb guides the cuboid against a set of “virtual fingers” (index, middle, ring). Contact points shift dynamically from ring–middle (frame 1) to middle–index (frames 3–4) as support from the ring finger decreases, and then shift back during regrasping (Krammer et al. [31]; Bullock & Dollar, [32])

In this study, the dynamic motor task observed with fMRI involved fine-tuned finger movements to manipulate a cuboid. The task mimics transitive motor actions found in everyday life, such as object positioning and somatosensory exploration [2, 29, 30]. It requires synchronous movements within grouped finger joints and asynchronous movements between groups, while the finger pads remain tightly adapted to the object’s surface [31]. The repetitive grasps and regrasps performed during a single manipulation closely resemble the acts of finger substitution in robotic dexterous manipulation, known as finger gaiting (FG, Fig. 1) [32]. In addition to the fMRI task, we used the Jebsen-Taylor hand function test and a tactile object recognition test to assess motor performance of the hand.

The motivation for studying hand paresis using a sequence of motor prehensions within the hand, characterized by grasping and regrasping within a single motor act, was the prospect of revealing fragmentation of coordinated and synergistic finger movements following sensorimotor cortex stroke [6]. For example, Xu et al. [33] found impaired dexterity in chronic stroke patients due to a loss of higher-order cortical control, while a tendency to finger flexion was due to an upregulation of subcortical control.

This was a longitudinal study in which we examined two cohorts of patients with acute ischemic sensori-motor stroke, characterized by hand paresis or plegia over the course of nine months. One cohort received escitalopram in the subacute stage of stroke for three months, whereas the other cohort received a placebo. The protocol proscribed four examinations, consisting of fMRI and behavioral evaluations, at the acute stage (ei), at baseline (e0) after final consent, at three months (e3) after which treatment with escitalopram was discontinued, and at nine months (e9), the conclusion of the study.

Our primary aim was to investigate whether interventional treatment with escitalopram in the first three months post stroke improves recovery from hand paresis compared to placebo, and evaluate the persistence of the effect until the end of the study at nine months. Our secondary objective was to evaluate whether escitalopram treatment at three months induces significant neuronal reorganization of motor execution, distinguishing between the escitalopram and placebo groups, and whether signs of deficient recovery remain evident in the placebo group at nine months. Our hypothesis was that escitalopram would improve recovery significantly, and that the improvement would persist over nine months.

## Subjects and methods

### Subjects

We prospectively recruited twenty-four patients aged between 59 and 85 years (median 78) at two comprehensive stroke centers (Departments of Neurology, University Hospital of Bern, Switzerland and Cantonal Hospital St. Gallen, Switzerland) from March 21, 2017 to August 13, 2020. Inclusion criteria were: (1) first ever stroke, (2) clinically significant contralateral hand plegia or paresis as main symptom and (3) involvement of the pre- and/or postcentral gyri, confirmed by diffusion-weighted imaging (DWI) and fluid-weakened inversion recovery (FLAIR) MRI scans. In addition, involvement of the frontal, parietal and opercular regions was accepted but not selected for. Main exclusion criteria included aphasia or cognitive deficits severe enough to preclude understanding of study purposes, prior cerebrovascular events and significant stenosis or occlusion of the carotid and intracranial arteries presented on magnetic resonance angiography. A detailed list of these criteria is given in Supplemental Information [Table ST1].

Stroke severity was assessed using the NIH Stroke Scale [34] and post-stroke disability with the Modified Rankin Scale (MRS). Cognitive function and psychological well-being of the patients were evaluated with the Mini-Mental State Examination (MMSE) [35], Beck’s Depression Inventory [36], and the Hamilton Depression Rating Scale [37]. Handedness was determined using the Edinburgh Handedness Inventory [38]. The International Standard Classification of Education (ISCED) coding characterized educational levels.

Of the twenty-four patients included in the study at the acute stage, occurring 1 to 14 days (median 4) after stroke (ei), one patient was unwilling to participate further because he felt too frail to continue; two patients withdrew from the study after the baseline examination (e0) due to the long and subjectively arduous traveling distances to the study centers. Of the remaining twenty-one patients, 19 were males and 2 females. Randomly selected, 12 patients composed the verum group and 9 the placebo group. The ages of the verum group ranged between 61 and 85 years (median 81) and those of the placebo group between 59 and 81 years (median 77). Treatment with escitalopram was discontinued in one patient of the verum subgroup due to fatigue and sedation after 67 days, i.e. before the scheduled three-month examination (e3). In one patient of the placebo group, the fMRI data exhibited severe artifacts, prohibiting image analysis. In summary, 21 patients completed the study, each providing complete behavioral and structural MRI datasets, including DWI, from the three examinations, e0 to e9, as well as complete fMRI datasets for 20 patients. For demographics and clinical characteristics at inclusion see Table 1.

**Table 1:** Baseline characteristics of the study cohort

Variable	Median (IQR)	
	Verum-group (*n* = 12)	Placebo-group (*n* = 9)
Age [in years]ª	81 (9)	77 (8)
Sex [n of men]	11	8
Education in ISCED coding [n]ª		
3 - Upper secondary education	1	0
4 - Post-secondary non-tertiary education	7	5
5 - Short-cycle tertiary education	4	3
6 - Bachelor or equivalent	0	0
7 - Master’s or equivalent	0	1
8 - Doctoral or equivalent	0	0
Mini-mental state examinationª	28 (3.3)^b^	28 (1)
Beck’s depression inventory [n]ª		
Minimal depression (0–13)	11	9
Mild depression (14–19)	1	0
Moderate depression (20–28)	0	0
Hamilton Depression Rating Scale [n]ª		
Normal (0–7)	11	8
Mild depression (8–13)	1	1
Edinburgh Handedness Inventory [n]ª		
Strongly right-handed (40–100)	11	9
Moderately right-handed (20–39)	1	0
Time since stroke onset at inclusion [days]ª	4 (2)	3 (3)
NIH Stroke Scale [n]ª	1 (2)	1 (1)
Modified Rankin Scale [n]ª	1 (1)	0 (1)
Hand paresis [n of right hand]ª	10	3
Power gripª	10 (19.5)	13 (10)
Precision gripª	4.3 (5.3)	7 (8.5)
Lesion size [in mm³], mean (SD)	9.5 (14.4)	7.6 (9.9)

Baseline demographic and clinical characteristics for the verum and placebo groups. Superscript “ª” denotes no statistically significant between-group differences (*p* > 0.05). Scalar values are presented as medians with ranges in brackets unless otherwise specified. Abbreviations: ISCED, International Standard Classification of Education; NIH, National Institutes of Health

Reference results for healthy subjects have been reported in detail recently [39]; this publication also describes the methodology applied in this study. The healthy control (HC) cohort consisted of 15 males and 13 females, two more than in the publication, ranging in age between 42 and 85 years (median 59). Comparisons for which age-matching was relevant selected the oldest 16 healthy subjects ranging in age between 55 and 85 years. (median 69). The healthy subjects were examined on a single visit.

## Methods

### Study design

The study was conducted as a prospective, longitudinal, multi-center, double-blind randomized trial. A computerized random-number generator determined patients to receive either escitalopram or a placebo pill over a 3-month period. The Institute of Pharmacy of the University Hospital Bern was responsible for a correct randomization procedure according to the guidelines of the ethics committee, and was actively involved in the allocation of the verum and placebo pills. The protocol prescribed four visits consisting of the comprehensive examinations detailed below. The initial visit (ei), planned for 3 to 5 days post-stroke, determined eligibility. The baseline examinations (e0), planned 14 ± 7 days post-stroke, were performed prior to randomization. Subsequent assessments included the first post-treatment examination (e3) at 90 ± 14 days post-stroke and a final evaluation (e9) at 270 ± 14 days post-stroke. The total study duration was planned to be approximately 265–267 days.

Randomization was performed in accordance with the guidelines of EudraLex (European Union Legislation) Volume 4, Annex 13. The Neuro Clinical Trial Unit (NCTU) of the University Hospital Bern supervised the randomization procedure and stored the randomization codes securely. They were inaccessible to the study staff and were disclosed only after the study’s completion, or in the case of a relevant side effect. Starting at visit e0, patients received escitalopram or placebo at a daily dose of 5 mg/day. The dose was increased by 5 mg per week until a maximum of 20 mg/day and continued at that dosage until visit e3. The medication was then reduced to 10 mg per day for 7 days and then tapered off. Adherence, medication compliance, side effects, and adequate occupational therapy were checked monthly on an outpatient basis or by telephone. During the first three months post-stroke (e0-e3), all patients received an average of 6.2 h of occupational therapy per month (SD = 4.5), specifically for hand paresis. Four patients continued therapy up to six months, averaging 6.8 h (SD = 3.4). All others stopped after three months.

The planned sample size of 50 patients and 20 healthy control subjects is based on data from a pilot study [40], calculated using the MATLAB software fMRI power, in order to achieve an effect size of 0.5 and power of at least 80% (see study protocol in repository, pp 46–48).

### Behavioral measures

#### Sensorimotor assessment

Sensorimotor functions in both hands, starting with the non-paretic hand, were assessed for elementary sensory and motor skills. Sensorimotor evaluations upon inclusion in the study (ei) and at e0 included pressure perception threshold (PPT) with graded monofilaments [41], grip strength measured with a Jamar hydraulic hand dynamometer for power grip and a pinch gauge between thumb and index finger for precision grip [42]. In addition to the FG test, manual dexterity and general hand function were assessed using the Jebsen-Taylor Hand Function Test (JTT) [43] and a tactile object recognition test (TOR) based on a standardized protocol with 30 everyday objects [44] throughout the study from baseline to month 9. In this study, we focused on five subtests of JTT: JTT subtest 1 comprised turning over five index cards (“turning cards”); JTT subtest 2 (PSO) picking small objects: two clips, two bottle caps and two coins with one hand, and dropping them into an empty can; JTT subtest 3 (“stack”) stacking four checkers on a board; JTT subtest 4 (“light”) lifting large light cans and placing them on a board; and JTT subtest 5 (“heavy”) lifting large heavy cans and placing them on a board. The maximum time to complete each subtest was set at 180 s [45]. These tests have shown high test-retest reliability in previous research; the two less reliable subtests, writing a standard sentence and simulated feeding were excluded [43, 46] due to (1) their complexity and involvement of cognitive components beyond specific motor actions, and (2) their emphasis on goal-directed tool use, such as with a spoon, which is associated with lower test-retest reliability [47, 48].

### Functional MRI task

The functional task was adapted from Roland and Mortensen [49] and evaluated in and established for clinical use [39] in normal volunteers. This task comprises tactual macroscopic object exploration featuring three subtasks: (1) fixation, in which participants fixated on a screen showing a resting hand holding a cube; (2) observation of a video showing a hand exploring the cube; and (3) manipulation, in which participants replicated the observed exploration. In an fMRI session, the three subtasks were repeated six times showing 3 male and 3 female hands, resulting in a total length of 7.2 min.

The task was designed to focus solely on sensorimotor activities, specifically dynamic finger movements around a cuboid shape, while aiming to minimize cognitive load. Participants were asked to replicate the sensorimotor action at the frequency of 1 Hz presented previously on the video. The granite cuboid, similar to that of Weisstanner et al. [40], had the dimensions 2.25 × 2.25 × 2.26 cm, a volume of 11.5 cm^3^, a density of 2.6 g/cm^3^, and a weight of 29.9 g. The manipulation involved consistent motor actions characterized by the dynamic interplay of the thumb and fingers of one hand encircling the cuboid in a steady, rhythmic manner at the rate of 1 Hz. The action started with the thumb moving transaxially against the ring finger. The synchronized movement of thumb and fingers led the cuboid to rotate anti-clockwise in the right hand and clockwise in the left hand [31]. The key measure was the object movement per motor act associated with the moving thumb against the opposed fingers. A close-up video was recorded from inside the MR scanner to evaluate FG during the fMRI scan. Analysis of the video provided the frequency of thumb manipulations, with six measurements per session averaged for the results.

### Neuroimaging

Initial MRI scans were performed on a 1.5T scanner following ischemic stroke. These routine scans were used to assess study eligibility and manually delineate lesions using DWI. All longitudinal study imaging sessions (e0, e3, e9) were conducted on 3T Siemens scanners (Skyra, *n* = 18; Prisma, *n* = 3) at the two study centers using fMRI and Modified Driven Equilibrium Fourier Transform (MDEFT) protocols with identical acquisition parameters. To ensure data comparability, two authors performed test scans on both scanners. No significant differences were observed in volumetric measures, cortical thickness determined with FreeSurfer v6.0 (http://surfer.nmr.mgh.harvard.edu), or functional activation patterns determined with SPM12 (Wellcome Trust Centre for Neuroimaging, University College London) implemented in MATLAB 2021a (MathWorks, Natick, USA), confirming compatibility of the scanner platforms.

### Diffusion weighted images (DWI)

At admission time (ei), DWI was performed using echo-planar imaging (EPI) on the 1.5 Tesla MRI scanners of the two centers. The majority of scans was acquired on a Siemens SymphonyTim system: repetition time (TR)  = 3943 ms, echo time (TE)  = 108 ms, 21 slices, slice thickness = 5.0 mm, matrix = 192 × 192, voxel size = 1.09 × 1.09 × 5.0 mm³, flip angle = 90°, scan time = 124 s. In three participants, DWI was acquired on a Siemens Avanto system using EPI with slightly different parameters: TR = 3000 ms, TE = 89 ms, 21 slices, slice thickness = 5.0 mm, matrix = 192 × 192, voxel size = 1.20 × 1.20 × 5.0 mm³, flip angle (FA)  = 90°, scan time = 57 s. The images were subsequently combined to generate a cohort lesion map.

### Structural images

T1-, T2-, and FLAIR-images and time-of-flight MR angiography were acquired at admission time (ei). The subjects were placed supine inside a 3 Tesla whole-body MR scanner (Siemens Magnetom Prisma, Siemens, Erlangen, Germany) with their heads immobilized in a standard head coil. Structural images were obtained using a MDEFT T_1_-weighted sequence with the following parameters: TR, 7.93 ms; TE, 2.49 ms; inversion time = 910 ms; FA 16°; field of view (FOV) and matrix dimensions 256 × 256; leading to an isotropic resolution of 1 mm. The total acquisition time was 823 s i.e. 13 min, 43 s.

### Functional MR-images

Functional images were acquired at the examination times e0, e3 and e9 using a T_2_*-weighted, echo-planar imaging–blood oxygen level-dependent (EPI–BOLD) sequence with parameters: TR, 3000 ms; TE, 30 ms; 49 slices; slice thickness, 2 mm; FA, 90°; FOV, 194 × 194 mm^2^; matrix dimensions 76 × 76; voxel resolution, 2.56 × 2.56 × 2 mm^3^; and acquisition time, 432 s.

### Statistical analysis

Differences in within-hand activities across the three examination points (e0, e3, and e9) were assessed using the Friedman test, a non-parametric alternative to a one-way repeated-measures ANOVA. Post hoc pairwise comparisons between time points (e0 vs. e3 and e3 vs. e9) were conducted using the Wilcoxon signed-rank test. Median dexterity hand activity at each time point was compared between verum and placebo subgroups using the Mann–Whitney *U* test.

### Preliminary quality control

Weight of the seven dexterity hand parameters. The seven dexterity hand parameters, FG, the JTT subtests, and the TOR, were initially analyzed as part of a primary statistical quality control procedure. This process aimed (1) to validate the contribution of each hand dexterity parameter in capturing performance deviations from HCs and in discriminating between the verum and placebo subgroups using Principal Component Analysis (PCA), and (2) to substantiate these findings, given the small patient sample, by applying permutation tests to compare subgroups.

Performance deviation from HCs. The seven behavioral measures (FG, TOR, JTT1–JTT5) were converted to *z*-scores using HC scores as reference; more negative z values for FG and more positive for the remaining scores indicated greater impairment.

Weight of the seven dexterity hand parameters using PCA. PCA was performed using the program MATLAB *pca.m*, and components were ranked by explained variance. Four patients who failed individual tasks were excluded, leaving 9 verum and 8 placebo participants. Components accounting for ≥ 80% of the variance (typically two) were retained for further analysis. Components differentiating groups (*p* < 0.05) were retained, and thresholds were estimated from score distributions. Variable reduction was performed according to the principle of parsimony (Occam’s razor) to define key parameters [Table ST3 in Supplemental Material].

Permutation analysis. For each set of scores at each time point e0, e3, e9, data of 12 verum and 9 placebo patients were coded (verum = 1, placebo = 0). Random permutations of this labelling were generated by partitioning 21 random numbers according to the 12/21 ratio. Median differences between groups were recalculated for each permutation, and the original median difference was compared with the randomized distribution to estimate the probability of observing the effect by chance [Table ST4 in Supplemental Material].

### Lesion analysis and summation maps

DWI volumes were motion-corrected and normalized to MNI space. Lesions were manually drawn per slice and subject using FreeSurfer v6.0. Lesion analysis included volumetric measurements performed with Mango v4.1 (http://www.mangoviewer.com). Statistical comparisons of lesion volumes were conducted using two-sample t-tests, comparing left- versus right-hemispheric lesions across all patients, as well as between the verum and placebo groups. Additionally, cluster-based analyses were performed using the Julich Histological Atlas v3.1 to characterize lesion distributions within predefined anatomical regions. Summation maps were generated using SPM12’s ImCalc tool, applying voxel-wise summation. The resulting summation map, saved in NIfTI format, was: (1) quality-checked using SPM’s Check Reg function, and (2) integrated into functional MRI activation maps to visualize the spatial relationship between lesioned and activated brain areas.

### Functional neuroimaging

Preprocessing. Statistical Parametric Mapping (SPM12) software was used for preprocessing and analysis of the functional MRI data. Preprocessing included the following steps: realignment, segmentation into grey matter, white matter, and cerebrospinal fluid, coregistration of functional images to structural images for each participant, normalization to the Montreal Neurological Institute (MNI) space, and spatial smoothing with a 6 mm full-width-at-half-maximum Gaussian kernel. DWI was utilized to create lesion masks of voxels affected by lesions, which were excluded in subsequent analyses.

Subject level analysis. For each participant, task sequences were defined by the onset and durations of each subtask during an acquisition, convolved with a canonical hemodynamic response function (HRF) to account for the delayed and dispersed nature of the BOLD signal in response to neural activity. Contrasts were computed to compare BOLD signals between subtasks: observation vs. fixation and manipulation vs. fixation.

Group level analysis. The statistical analysis proceeded in two stages: (1) A voxel-wise omnibus *F*-test using one-way ANOVA was applied to assess differences among the contrasts of the verum group (VG), placebo group (PG), and HC group across all three visits: e0-e9. Activation clusters of high variance were identified by applying a family-wise error (FWE) correction with p_FWE_ < 0.05 and a minimum cluster size of k > 20 voxels. Generalized eta squared (η²G) was computed for each ANOVA to quantify group effect size. (2) Tukey HSD post-hoc correction was performed to limit the FWE rate to a level of *p* < 0.05 for any number of pairwise comparisons, thereby localizing significant differences between the patient subgroups and between the patient subgroups and the normal HCs. All results are reported with 95% confidence intervals (CIs). The MNI coordinates specify the center of gravity of the clusters. Localization of lesions and regions of interest was performed using the Julich Cytoarchitectonic Atlas 3.1 (EBRAINS, Siibra). Additionally, we estimated marginal means (EMMs) representing the model-adjusted average global BOLD signal during manipulation for each subgroup, allowing comparison of patients and healthy controls at baseline and pairwise comparison of contrasts between verum and placebo subgroups from a generalized estimating equation (GEE) repeated-measures model.

## Results

### Clinical course

The comprehensive follow-up examinations were done in accordance with the planned schedule. Median time post-stroke to examinations was: for e0 17 days (range 7–31), for e3 92 days (range 67–127), and for e9 273 days (range 257–355). The number of days post stroke was not statistically different for visits between the verum and placebo groups. The average duration of treatment, including the patient who discontinued treatment prematurely, was 72.5 days (range 36 to 120) for the verum group and 84 days (range 64 to 88) for the placebo group; the difference is insignificant: z = 0.75, *p* > 0.05. A total of 12 patients presented lesions in the left hemisphere and, consequently, used their right hand for the task during the fMRI, while 8 patients presented lesions in the right hemisphere and used their left hand.

The baseline measures were evenly distributed across the group (Table 1). There was significant moderate to severe hand paralysis as determined by elementary sensorimotor tasks such as power grip, precision grip and pressure perception threshold; there was no associated spastic hand flexion at all. At study inclusion (ei), NIHSS (National Institutes of Health Stroke Scale) scores were low: median 1 (range 0–6); in particular, the motor score for the arm, including ataxia, remained stable from baseline (e0) until the end of study, with a median score of 0 (range 2). Other scores were also low: mRS: 1 (0–3), Beck Depression Inventory: 4 (1–14), Hamilton Depression Scale: 0 (1–10), MMSE: 1 (0–6). No meaningful trends emerged in these scales over time.

### Behavioral measures

**Fig. 2:**
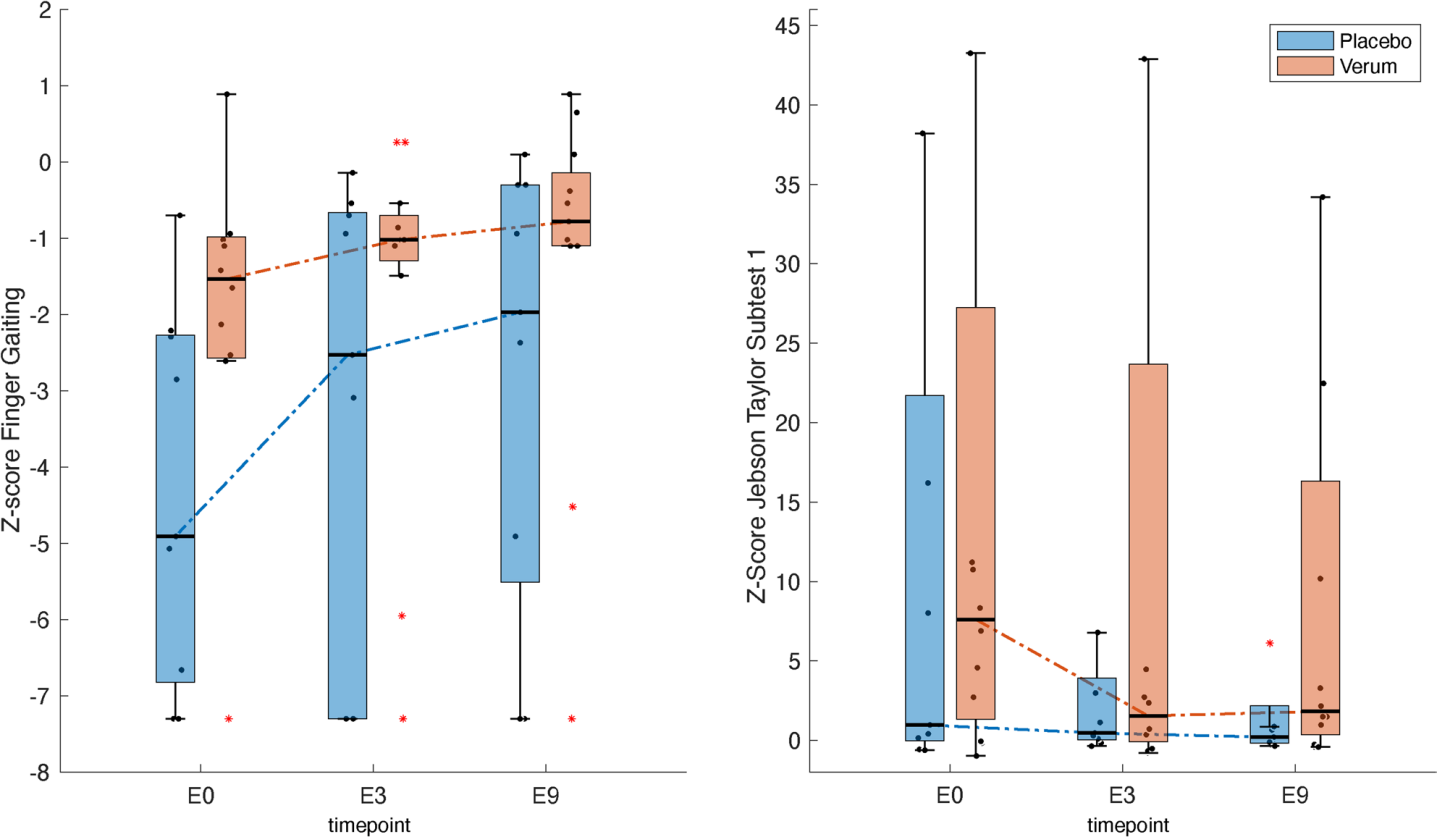
Nine-month recovery trajectories for finger gaiting and JTT1. Distribution of z-scores for the verum group (red) and the placebo group (blue) over the entire study period. They are shown in a box-whisker plot, which indicates the minimum value, the first quartile, the median, the third quartile, and the maximum value. Data points that lie far outside the range of the whiskers are shown as individual red stars. A z-score of 0 corresponds to the average performance of healthy control subjects. For JTT1, extreme values (z ≈ 60; task not completed, two for verum and one for placebo at e0 and e3, one each for both subgroups at e9 were omitted for clarity

Group-level changes. According to the Friedman test and post hoc Wilcoxon analyses (Table 2), the verum group demonstrated a trend toward improvement in FG frequency, while JTT1 (card turning) showed a similar tendency. Figure  2 illustrates convergence of patient performance toward the HC median intervals, suggesting partial normalization of FG frequency and JTT1 completion time. However, substantial baseline differences between subgroups hinder clear interpretation.

**Table 2:** Deviation from healthy controls across seven hand dexterity measures

Variable	Median (Range)				
	Patients (*n* = 21) Baseline (e0)	Patients (*n* = 21) 3 months (e3)	Patients (*n* = 21) 9 months (e9)	Healthy Controls (*n* = 28)	Kruskal Wallis [H]
Finger gaiting [s^-1^]	0.59 (1.03)**	0.79 (0.95)**	0.78 (1.03)**	0.92 (0.2)	30.8, *p* < 0.0001
JTT 1 [s]	19.3 (176)**	7.3 (175.6)*	7.8 (174.9)*	5.6 (2.5)	19.2, *p* < 0.0005
JTT subtest 2 [s]	8.1 (176.1)	7.9 (174.7)	7.9 (174.6)	6.3 (1.9)	5.52 ^ns^
JTT subtest 3 [s]	6.9 (176.5)	5.8 (60.3)	5.8 (70.1)	5.3 (1.5)	5.31 ^ns^
JTT subtest 4 [s]	4.8 (177.1)	4 (25.8)	4 (49.1)	4 (0.38)	5.01 ^ns^
JTT subtest 5 [s]	4.5 (177)	3.9 (23.6)	3.7 (13.1)	3.6 (0.53)	9.62, *p* < 0.02
TOR [n]	26 (30)**	29 (25)	30 (30)	29 (2)	22.3, *p* < 0.0001

Group differences in patient performance relative to healthy controls (HCs) across seven dexterous hand tasks. Nonparametric statistics were used: Kruskal–Wallis for omnibus testing followed by Mann–Whitney *U* tests. Age-independent parameters were compared to the full HC sample (*n* = 28; 42–85 years), whereas age-dependent parameters were compared to an age-matched HC subset (*n* = 16; 60–85 years). A two-sided significance threshold of *p* < 0.05 was applied, with Bonferroni correction for multiple comparisons (*p* < 0.005 indicated by *, *p* < 0.01 by **). FG and JTT1 showed the largest long-term deviations (e0–e9). The transient deviation in TOR at baseline reflects tactile pseudoagnosia due to severe paresis limiting object exploration in 4 out of 5 cases

Comparison with healthy controls. Neither FG nor JTT1, both reflecting within-hand coordination, exhibited age dependence in the HC cohort (ages 42–85 years). By contrast, the remaining JTT subtests, which require combined hand and arm movements, demonstrated clear age dependency. A similar age-related pattern was observed for TOR, which involves grasping, regrasping, and object recognition [Table ST2 in Supplemental Material].

Table 2. Deviation from healthy controls across seven hand dexterity measures Performance deviation from HCs. FG and JTT1 were compared with the entire HC cohort (ages 42–85 years), whereas the remaining age-dependent measures were compared with an age-matched HC sub-cohort (*n* = 16, ages 60–85 years). Notable longitudinal changes were observed for FG and JTT1 between baseline and month 9. These two parameters were the only ones among the seven dexterity measures that differed significantly from the HC reference (right hand) over the long-term course (e0-e9) and survived Bonferroni correction for multiple comparisons. Performance among HCs was side-independent across all tests; therefore, patient performance was evaluated relative to right-hand data from HCs. Other parameters showed minimal or no group differences throughout the study, except for TOR, which differed significantly at baseline only.

Weight of the seven dexterity hand parameters using PCA. Among the seven behavioral measures, FG and JTT1 emerged as the primary discriminators between verum and placebo groups, identified via variance-based ranking and parsimony. At e9, the second PCA component (FG/JTT1) explained 37% of the variance and significantly distinguished verum from placebo participants (*p* < 0.015). Receiver Operating Characteristic (ROC) analysis indicated moderate classification performance (true positive rate: 89%; false positive rate: 43%; AUC = 0.70) [ST3 in the Supplemental Material].

Permutation analysis. Permutation testing with > 10,000 randomizations (12 verum, 7 placebo) revealed a significant median difference for FG at baseline (e0), but not at later time points (e3, e9). For JTT1, no significant differences were detected across time. Consequently, the apparent significance of FG from e0 to e3 observed in the original dataset was not confirmed, whereas FG from e3 to e9 and JTT1 from e0 to e3 remained significant. Detailed median differences and likelihood levels are provided in Table 3. In summary, FG improved between months 3 and 9, and JTT1 improved between baseline and month 3, with both effects remaining robust under permutation testing [Table ST4 in Supplemental Material].

**Table 3:** Time courses of finger gaiting and JTT subtest 1 in the patients subgroups

Task	Subgroup	Baseline (e0)	3 Months (e3)	9 Months (e9)	Wilcoxon signed rank test [W]		Friedman test [csq]
		Median (IQR)			e0 vs. e3	e3 vs. e9	
FG [s^-1^]	Verum	0.73 (0.20)	0.79 (0.05)	0.82 (0.11)	-51, ^ns#^	-54*	12.0, *p* < 0.01*
	Placebo	0.3 (0.55)	0.60 (0.83)	0.67 (0.88)			4.06 ^ns^
MW		z = 1.7 ^ns^	z = 0.9 ^ns^	z = 1.1 ^ns^			
Median difference between permuted versus real data in z-scores and time units (in brackets)		2.49 (0.31)**	1.11 (0.17)^ns^	0.88 (0.12)^ns^			
JTT subtest 1 [s]	Verum	20.7 (33.4)	8.9 (27.1)	9.5 (24.5)	64*	48 ^ns^	8.2, *p* < 0.05
	Placebo	7.8 (31.2)	6.8 (5.6)	6.3 (1.9)			1.89 ^ns^
MW		z = 0.53 ^ns^	z = 0.14 ^ns^	z = 1.07 ^ns^			
Median difference between permuted versus real data in z-scores and time units (in brackets)		4.49 (8.52) ^ns^	0.32 (2.13)^ns^	0.73 (1.41)^ns^			

Comparison of median differences between the verum and placebo groups based on measured values and 10,000-iteration permutation samples. Permuted values are presented as z-converted scores shown in Fig. 2, in brackets the corresponding time units. “#” indicates that significance observed in the Mann–Whitney test was not supported by permutation testing. * *p* < 0,05 corrected for multiple comparisons according to Bonferroni (the exact value for FG from e3 to e9 is *p* < 0.017, for JJT 1 from e0 to e3 is *p* < 0.0048). ** *p* < 0.05 also corrected for multiple comparisons (exact value *p* < 0.02)

### Neuroimaging

#### Lesion analysis and summation maps

DWI confirmed that all patients had lesions involving the precentral gyrus, postcentral gyrus, or dorsal premotor cortex, consistent with the inclusion criteria. Cluster analysis identified eight lesion clusters (four per hemisphere) covering on average 94% of the total lesion volume [Figure SF1, Supplemental Material]. Mean lesion size did not differ significantly between hemispheres (left: 9.8 ± 14.1 mm³; right: 7.3 ± 10.3 mm³; *t*_(19)_ = 0.78, *p* = 0.43), nor between treatment subgroups (verum: 9.5 ± 14.3 mm³; placebo: 7.6 ± 9.9 mm³). A full overview of lesion distributions, including regions beyond the inclusion criteria, is provided in Table ST5 [Supplemental Material].

Summation maps revealed lesion overlap in 2–6 patients within the hand area of the precentral and postcentral gyri and the superior parietal lobule. Overlap with fMRI activation maps showed that lesioned regions lay adjacent to, and in some cases intersected, the main activation clusters, particularly in primary sensorimotor cortex. The left-hemispheric map encompassed 6.2 ± 8.9 mm³ (64% of the average lesion volume), and the right-hemispheric map 7.0 ± 9.9 mm³ (96%), indicating minimal side differences.

### fMRI findings

The fMRI results revealed two main patterns (Table 4): (1) significant differences between the patient subgroups, suggesting a potential treatment effect of escitalopram, and (2) significant differences between both patient subgroups and HCs, indicating disease-related alterations at multiple time points.

**Table 4:** Regions showing significant between-group differences in BOLD activity during motor execution

Baseline (e0)		F-Value _(2, 41)_	*P*-Value _FWE_	MNI coordinates			HCs vs. placebo-group	HCs vs. verum-group	Placebo-group vs. verum-group
				X	Y	Z			
	R PCG, area 6v3, 6v2	24.52	< 0.001	59	7	31	-2.61* [-5.20, -0.02]	-2.85* [-5.12, -0.59]	-0.25 ^ns^ [-3.26, 2.77]
3 Months (e3)									
	R PCG, area 6v3	29.65	< 0.001	57	7	30	0.52 ^ns^ [-1.78, 2.82]	-2.37* [-4.51, -0.24]	-2.90* [-5.58, -0.21]
	L frontal OP, area Op6, post inf BA44	21.71	0.001	-50	6	4	1.88** [0.62, 3.13]	-0.09 ^ns^ [-1.26, 1.08]	-1.97** [-3.44, -0.50]
	L putamen	25.01	< 0.001	-30	-19	7	0.54 ^ns^ [-0.04, 1.11]	-0.15 ^ns^ [-0.68, 0.38]	-0.69* [-1.36, -0.02]
	L anterior insula area Id6, 7, 8	19.76	< 0.003	-31	19	10	1.59*** [0.73, 2.44]	0.16^ns^ [-0.63, 0.96]	-1.42** [-2.41, -0.43]
9 Months (e9)									
	L thalamus, medial-dorsal nucleus and anterior intraluminal nuclei	45.16	< 0.001	-20	-20	10	-0.80** [-1.38, -0.22]	0.13 ^ns^ [-0.37, 0.64]	0.93** [0.26, 1.60]

Significant group differences in BOLD activation across predefined regions of interest. Abbreviations: PCG, precentral gyrus; OP, operculum; BA, Brodmann area; R, right; L, left. All *p*-values are family-wise error (FWE)-corrected at 0.05. Post-hoc comparisons are adjusted using Tukey’s HSD. Coordinates represent the Montreal Neurological Institute (MNI) centre of gravity for each cluster. Significance levels: *p* < 0.05 (*), p < 0.01 (**), p < 0.001 (***), ns = not significant

### General effects across conditions

**Fig. 3:**
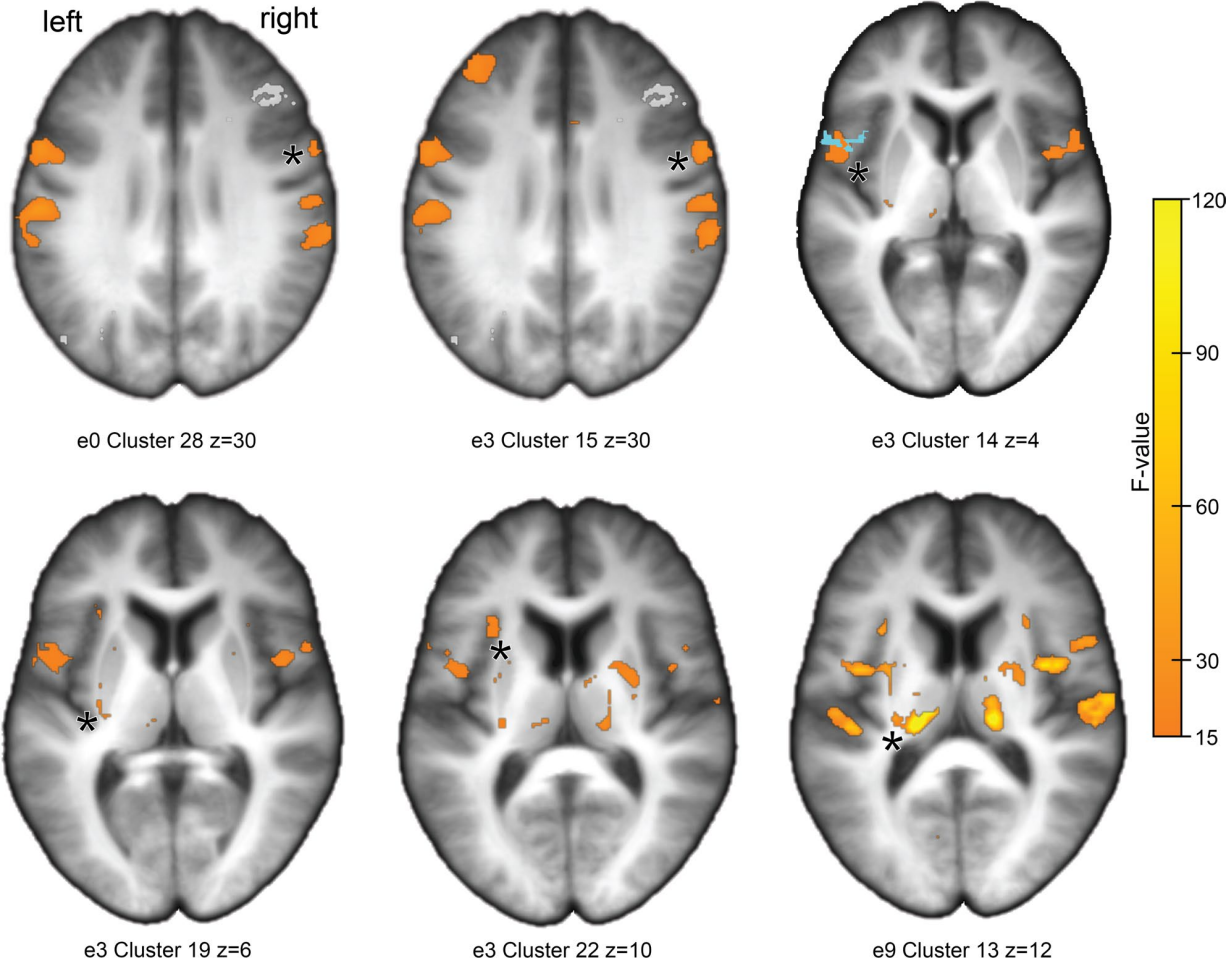
Group differences in BOLD activity during the manipulation block. Voxel-wise variance differences (F-test) displayed on group-average T1-weighted images (MRICro; Rorden & Brett [85]). Panels show contrasts among verum, placebo, and healthy control (HC) groups at baseline (e0), 3 months (e3), and 9 months (e9). Significant post-hoc Tukey HSD effects (*p* < 0.05) are marked with asterisks. At e0, both patient groups show increased activation in right ventral premotor cortex (subareas 6v3/6v2) relative to HCs. At e3, the verum > placebo contrast reveals increased activation in right 6v3 and left frontal operculum (OP6 and posterior-ventral BA44; BA44 ROI in blue per Clos et al., [69]). At e3, verum > placebo differences appear in left posterior putamen, left inferior frontal gyrus, and left anterior insula. At e9, placebo > verum activation emerges in the left mediodorsal thalamus Semi-transparent grey overlay (upper row, left and middle panel) depicts a composite lesion map of two patients involving the right dorsolateral prefrontal cortex

During the observation phase of the fMRI task, no significant group differences in BOLD activation were detected, neither between the verum and placebo subgroups nor between either subgroup and HCs, based on the specified contrasts. In contrast, the active manipulation phase yielded significant between-group differences in BOLD activation, representing the study’s primary neural findings (Fig. 3).

**Fig. 4:**
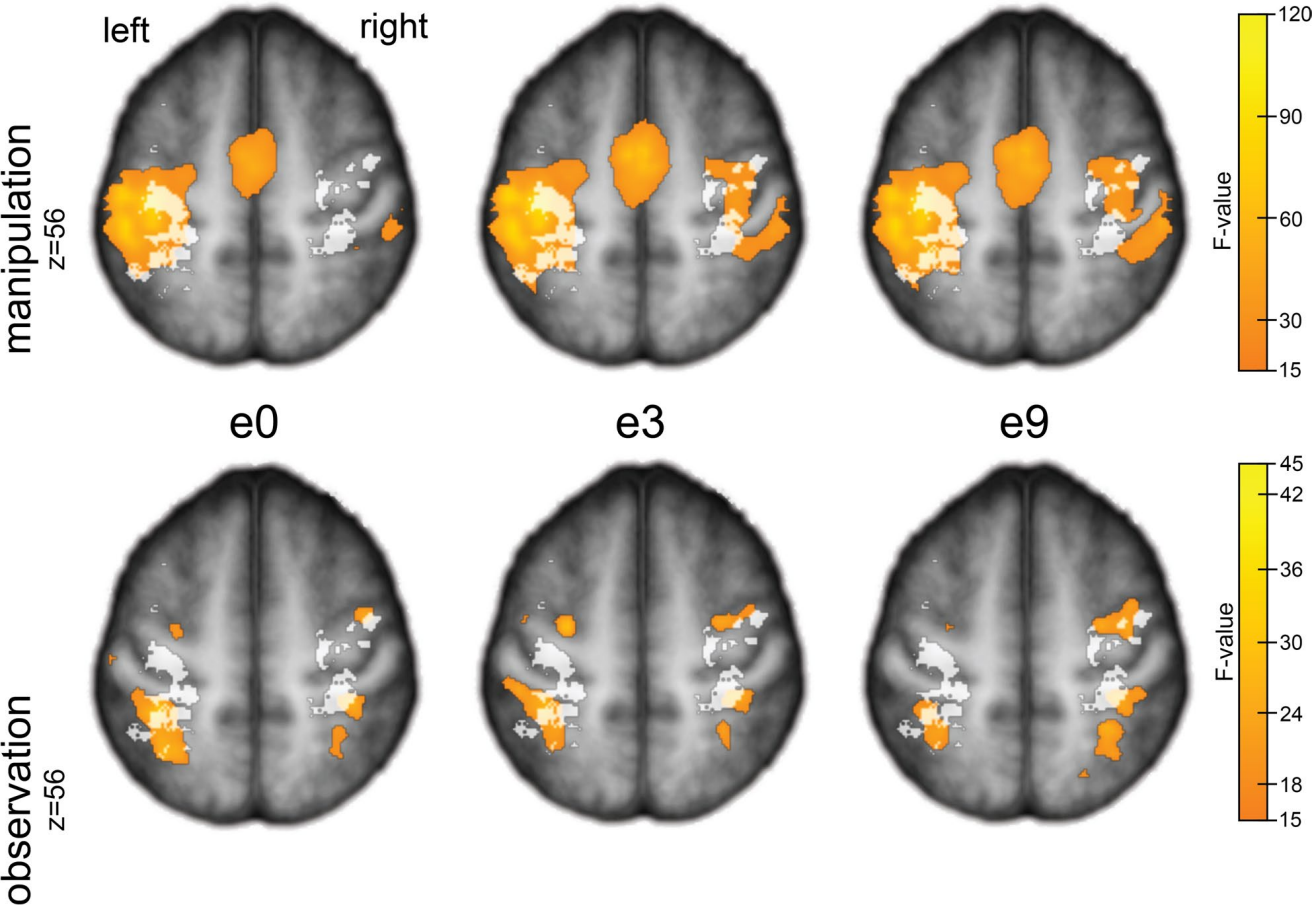
BOLD activity during manipulation versus observation. Voxel-wise variance differences (one-way ANOVA) shown on group-average T1-weighted images (MRICro). Rows compare motor execution (top) and action observation (bottom) at e0, e3, and e9 across verum, placebo, and HC groups. All panels show the same axial slice (z = 56) for direct comparison The grey semi-transparent overlay shows the cumulative lesion map (voxels lesioned in 2–6 patients), illustrating the spatial relationship between structural damage and functional activation. During motor execution at e0, BOLD activity appears reduced in primary sensorimotor hand cortex but preserved in dorsolateral premotor cortex (6d1) during the preceding observation phase

A detailed summary of high-variance BOLD activations across groups and conditions is provided in Supplemental Tables ST8 and 9. These also include ambiguous post hoc Tukey HSD results, which indicated differences only between one patient subgroup and HCs, thus limiting conclusive interpretation regarding a specific treatment effect or subgroup-defining characteristic. Figure 4 illustrates regions of high BOLD signal variability within the dorsal sensory pathway during both observation and manipulation conditions.

Pairwise contrasts of GEE-derived EMMs, adjusted for covariates, revealed distinct temporal trajectories: EMMs were reduced at baseline in both verum and placebo groups, normalized progressively in the verum group up to e9, and showed a sharp transient increase at e3 followed by a moderate decline at e9. Pairwise contrasts from the GEE model revealed a significant difference between groups at 3 months (e3), despite comparable levels at baseline and at 9 months, indicating a transient divergence in global BOLD activity between the verum and placebo subgroups. A significant BOLD difference between subgroups was observed at e3 [Tables ST6 and 7 in Supplemental Material].

Baseline (e0). During motor execution, both the verum and placebo groups exhibited significantly greater BOLD activation than HCs in subareas 6v3 and 6v2 of the ventral precentral gyrus (MNI [7, 31, 59]), extending into Brodmann area 44 (*F*_(2, 41)_ = 24.52, *p* < 0.001, η²G = 0.23).

Three months (e3). During the active manipulation phase, the verum group showed a pronounced increase in BOLD response compared both with the placebo and HC group in subarea 6v3 of the right ventral precentral gyrus (MNI [7, 30, 57]; *F*_(2, 41)_ = 29.65, *p* < 0.001, η²G = 0.17). Normalization of BOLD activity toward HC levels in the verum group was observed in the left frontal operculum (MNI [−50, 6, 4]), extending into the posterior–ventral portion of BA 44 (*F*_(2, 41)_ = 21.71, *p* < 0.001, η²G = 0.25), the left anterior insula (MNI [−31, 19, 10]; *F* = 19.76, *p* = 0.003, η²G = 0.32), and the left posterior putamen (MNI [−28, −19, 7]; *F*_(2, 41)_ = 25.01, *p* < 0.001, η²G _(2, 41)_= 0.14).

Nine months (e9). At follow-up, BOLD activation was significantly greater in the placebo group than in the verum group within the left mediodorsal thalamus (MNI [−20, −20, 10]; *F*_(2, 41)_ = 45.16, *p* < 0.001, η²G = 0.25), a key node of the subcortical-cortical loop connecting to the dorsolateral prefrontal cortex.

Most importantly in a direct comparison of the patient subgroups, the relevant ROIs of the verum group showed normalized BOLD values at e3, while the placebo group showed significantly decreased BOLD values at these sites. Conversely, the significant ROI of the verum group also showed a normalized BOLD value at e9, while the placebo group showed significantly increased BOLD values at this point.

## Discussion

This longitudinal study examined a carefully selected cohort of early post-stroke patients with pronounced sensorimotor hand paresis or plegia due to infarction in the precentral and/or postcentral sulcus. Importantly, the two treatment groups exhibited lesions of comparable extent in both hemispheres, controlled baseline functional status across behavioral and fMRI measures, and no confounding cognitive or affective impairments as verified by standardized assessments. The stringent inclusion criteria, built on clinical characteristics and acute-stage DWI lesion localization, constitute a major methodological strength. Within such a homogeneous cohort, the use of highly specific functional tests is crucial for detecting subtle deficits in manual dexterity and increasing the sensitivity of longitudinal behavioral and fMRI assessments [50].

### Conceptual framework and task design

Imitation, grounded in associative sequence learning [51], is a core mechanism in neurorehabilitation. Our study used a combined observation–manipulation paradigm involving graspable objects, introduced by explicit instruction. Evidence from a Cochrane review indicates that action observation therapy can improve upper-limb motor function after stroke, albeit with modest effect sizes [52], and Ertelt et al. [53] emphasized that observation followed by imitation forms the central mechanism of effective video-based training. Accordingly, our paradigm integrated an initial observational phase with subsequent active execution, both under structured supervision. These phases rely on coordinated multimodal (visual and haptic) sensory processing [54], enabling detection of modality-specific impairments.

The dynamic grasp–regrasp task used in the fMRI sessions, requiring finely graded finger sequencing, has proven highly sensitive for assessing long-term dexterity recovery after stroke [2, 55]. By isolating prototypical motor sequences from additional cognitive demands such as object exploration [31], the task yielded a purer measure of dexterity comparable to robotic “finger gaiting”, where alternating fingers support grasp stability as joint limits are reached [32, 56]. Performed at 1 Hz, the task lies well within the sensorimotor capacity of older adults; young adults typically reach ~ 1.3 Hz [57] and trained musicians 2–2.5 Hz [58]. Thus, the age-independence of finger gaiting observed in this study likely reflects the moderate frequency demands.

### Behavioral findings

Each Jebsen-Taylor Test (JTT) subtest was analyzed independently to preserve domain specificity. JTT1 reflects fine multi-finger coordination; JTT2 and JTT3 assess combined finger and targeted arm movements; JTT4 and JTT5 measure precision and power grip under target-directed conditions. A global score would have reduced sensitivity by collapsing across heterogeneous motor domains [59].

The focus on FG and JTT1 was theoretically and empirically grounded: measures with high initial deviation and intra-individual variability best capture meaningful recovery [2]. These parameters emerged as the principal discriminators between verum and placebo groups, identified through PCA and ROC analysis, both indicating discriminatory performance above chance. Because these measures tap within-hand dexterity rather than proximal limb movements, they provide sensitive markers of fine-motor recovery.

Permutation-based robustness analysis further strengthened the reliability of these findings. A significant median difference in FG at baseline (e0) was attributed to subgroup imbalance rather than a treatment effect, as the later time points (e3 and e9 for FG; all time points for JTT1 met quality-control criteria. Two effects remained significant under permutation: FG from e3 to e9 (with equal baselines) and JTT1 from e0 to e3. In contrast, FG at e0 did not share equivalent baselines and likely reflected random variation. These findings support the internal validity of the behavioral results and align with stroke rehabilitation studies employing permutation-based inference for small-sample upper-limb outcomes [60–63]. Nonetheless, the modest sample size and nonparametric data structure call for cautious interpretation and replication in larger, more diverse cohorts.

### Neuroimaging findings

At baseline, both patient groups displayed enhanced BOLD activation in the right ventral precentral gyrus (subarea 6v3), extending into BA44, relative to HCs. This region corresponds to the human analogue of macaque area F5c, implicated in implicit motor recognition [64, 65]. Such hyperactivation may reflect compensatory adjustments for reduced readiness of fine-motor execution, further supported by diminished primary sensorimotor cortex activity but preserved engagement of dorsolateral premotor cortex (6d1) during the observation phase [66, 67].

**Fig. 5:**
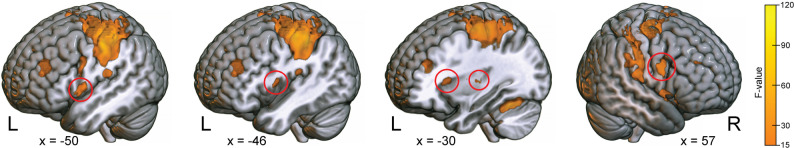
Significant hemispheric activations in the verum group after treatment. Surface and sagittal renderings of group-average T1-weighted images (MRICro) showing regions of high variance (warm colors) identified by one-way ANOVA at e3. Post-hoc Tukey contrasts highlight verum-dominant effects (red outlines) Significant BOLD activations include the posterior-ventral BA44 compartment and OP6 of the left inferior frontal gyrus, left posterior putamen, left anterior insula, and right ventral premotor cortex

At three months, the verum group exhibited marked hyperactivation in right 6v3 compared with placebo - consistent with the known right-dominant specialization of this region and the homologous macaque area 5Fc [68]. Partial recovery of dynamic finger control was evident in the right primary motor cortex. In the left hemisphere, BOLD normalization toward HC levels appeared within a premotor–opercular–striatal network encompassing the frontal operculum (OP6, ventral BA44), anterior insula, and posterior putamen (Fig. 5). Activation in the frontal operculum [69, 70] aligns with its role in visually guided manual action monitoring [71–73]. The inferior frontal gyrus exhibits mirror-neuron properties: its dorsal compartment activates during both observation and execution, while the ventral compartment - engaged here - responds selectively during execution [74]. OP6, characterized by large pyramidal neurons, likely mediates sensorimotor imitation through interactions with the anterior insula and posterior putamen [70]. Meta-analytic connectivity modelling of BA44 demonstrates co-activation of the insula, putamen, and posterior-ventral BA44 [69].

The anterior insula, a hub of executive control [75, 76], interacts with dorsolateral prefrontal and posterior parietal regions within the central executive network [77]. Its engagement here may reflect emerging volitional control of sensorimotor actions [78]. The posterior putamen, which receives input from supplementary motor and somatosensory cortices, participates in basal ganglia–thalamo–cortical circuits [79]. Its activation suggests a shift from associative to sensorimotor domains, consistent with automatization of performance [80, 81].

By nine months, increased activation in the left mediodorsal thalamus was observed in the placebo group, indicating compensatory recruitment of the dorsolateral prefrontal loop within the basal ganglia–thalamo–cortical network [79, 82]. This pattern, associated with attentional monitoring in chronic impairment [83], contrasted with efficient, normalized activation in the verum group.

Overall, at three months the verum group showed normalized BOLD activity in premotor–opercular–striatal regions (OP6, BA44, anterior insula, posterior putamen) and increased activation in right 6v3 compared with placebo, accompanied by superior dexterity performance on JTT1. By nine months, the verum group maintained normalized BOLD activity with superior performance on FG, suggesting more efficient neural recruitment, whereas the placebo group displayed thalamic overactivation consistent with compensatory effort [83]. The distinct temporal trajectories in behavioral and regional neural measures are compatible with a group × time interaction, reflecting differences in both the rate and phase of recovery between groups. Importantly, this transient divergence was mirrored at the global level: pairwise contrasts from the GEE model demonstrated a significant between-group difference only at three months, despite comparable baseline and nine-month values, indicating differential evolution of neural activity over time. Global BOLD hyperactivation at month three in the placebo subgroup was further supported by EMMs from the repeated-measures GEE model.

### Methodological considerations and limitations

The primary limitation of this study is the modest sample size, dictated by stringent inclusion criteria that ensured a homogeneous and clinically well-characterized cohort. While this enhances internal validity, generalization to the broader stroke population must be cautious. Additional strengths include the targeted selection of hand dexterity tasks to isolate specific sensorimotor deficits and the use of an fMRI paradigm optimized for fine-motor control [84]. Large local BOLD effects in the verum group (η²G > 0.14) persisting to nine months highlight the robustness of the observed neural changes. Nonetheless, heterogeneities in stroke pathology - such as flexion bias or spasticity due to subcortical injury not present in our patient cohort - may influence task performance and necessitate individualized measurement strategies [33]. This work provides a mechanistic foundation and justification for future large-scale trials investigating the therapeutic potential of SSRIs in post-stroke motor recovery.

## Supplementary Information

Below is the link to the electronic supplementary material.

Supplementary material 1.

## Data Availability

The datasets generated during the current study and study protocol are available in the Open Science Framework (OSF) repository at [https://osf.io/k9jqb](https:/osf.io/k9jqb) (DOI [10.17605/OSF.IO/K9JQB](https:/doi.org/10.17605/OSF.IO/K9JQB) ).
