# Correction: Escitalopram promotes recovery from hand paresis in cortical sensori-motor stroke: a randomized, double-blind, placebo-controlled longitudinal study

**DOI:** 10.1186/s12984-026-01964-1

**Published:** 2026-04-06

**Authors:** Vanessa Vallesi, Werner Krammer, Andrea Federspiel, John H. Missimer, Manuela Pastore-Wapp, Georg Kägi, Roland Wiest, Bruno J. Weder

**Affiliations:** 1 Support Centre for Advanced Neuroimaging (SCAN), Institute for Diagnostic and Interventional Neuroradiology, Inselspital, Bern University Hospital, University of Bern, Bern, Switzerland; 2 Advanced Imaging Research (AIR) Group, Swiss Paraplegic Research, Nottwil, Switzerland; 3 Department of Neurology, Kantonsspital St.Gallen, St.Gallen, Switzerland; 4 Laboratory of Biomolecular Research, Paul Scherrer Institute, Villigen, Switzerland; 5 Gerontechnology & Rehabilitation Group, ARTORG Center for Biomedical Engineering Research, University of Bern, Bern, Switzerland; 6 Department of Neurology, Bern University Hospital, Inselspital, University of Bern, Bern, Switzerland

**Correction: Journal of NeuroEngineering and Rehabilitation (2026) 23:83**

10.1186/s12984-026-01888-w

In this article [1], the graphics relating to Figs. 4 and 5 captions had been interchanged; the figure(s) should have appeared as shown below.

The Incorrect and correct figures with the captions are given below.

The original article has been corrected.

Incorrect Fig. 4

**Figure:**
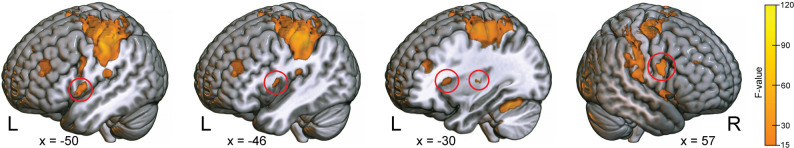
**Fig. 4** BOLD activity during manipulation versus observation. Voxel-wise variance differences (one-way ANOVA) shown on group-average T1-weighted images (MRICro). Rows compare motor execution (top) and action observation (bottom) at e0, e3, and e9 across verum, placebo, and HC groups. All panels show the same axial slice (z = 56) for direct comparison The grey semi-transparent overlay shows the cumulative lesion map (voxels lesioned in 2–6 patients), illustrating the spatial relationship between structural damage and functional activation. During motor execution at e0, BOLD activity appears reduced in primary sensorimotor hand cortex but preserved in dorsolateral premotor cortex (6d1) during the preceding observation phase

Correct Fig. 4

**Fig. 4:**
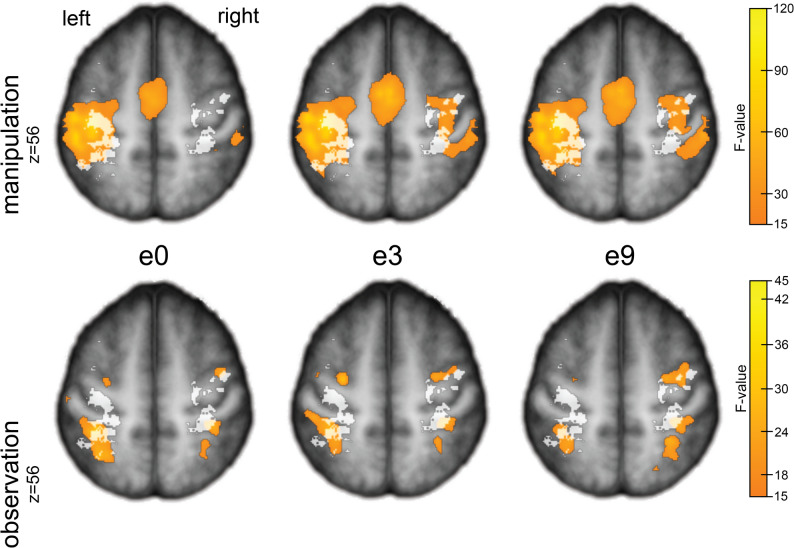
BOLD activity during manipulation versus observation. Voxel-wise variance differences (one-way ANOVA) shown on group-average T1-weighted images (MRICro). Rows compare motor execution (top) and action observation (bottom) at e0, e3, and e9 across verum, placebo, and HC groups. All panels show the same axial slice (z = 56) for direct comparison The grey semi-transparent overlay shows the cumulative lesion map (voxels lesioned in 2–6 patients), illustrating the spatial relationship between structural damage and functional activation. During motor execution at e0, BOLD activity appears reduced in primary sensorimotor hand cortex but preserved in dorsolateral premotor cortex (6d1) during the preceding observation phase

Incorrect Fig. 5

**Figure:**
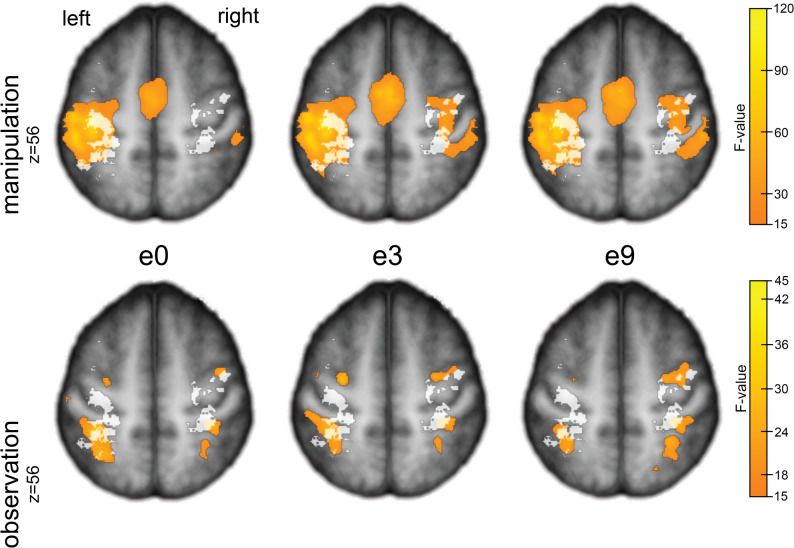
**Fig. 5** Significant hemispheric activations in the verum group after treatment. Surface and sagittal renderings of group-average T1-weighted images (MRICro) showing regions of high variance (warm colors) identified by one-way ANOVA at e3. Post-hoc Tukey contrasts highlight verum-dominant effects (red outlines) Significant BOLD activations include the posterior-ventral BA44 compartment and OP6 of the left inferior frontal gyrus, left posterior putamen, left anterior insula, and right ventral premotor cortex.

Correct Fig. 5

**Fig. 5:**
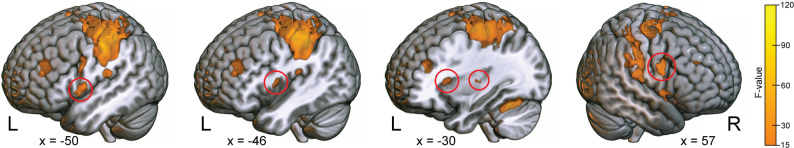
Significant hemispheric activations in the verum group after treatment. Surface and sagittal renderings of group-average T1-weighted images (MRICro) showing regions of high variance (warm colors) identified by one-way ANOVA at e3. Post-hoc Tukey contrasts highlight verum-dominant effects (red outlines) Significant BOLD activations include the posterior-ventral BA44 compartment and OP6 of the left inferior frontal gyrus, left posterior putamen, left anterior insula, and right ventral premotor cortex.
