# Supplementary material for: Escitalopram promotes recovery from hand paresis in cortical sensori-motor stroke: a randomized, double-blind, placebo-controlled longitudinal study
Source: J Neuroeng Rehabil. 2026 Jan 31;23:83. doi: 10.1186/s12984-026-01888-w (PMC12952094; doi:10.1186/s12984-026-01888-w)
Supplement: Supplementary file 1 — Supplementary material 1. [file 12984_2026_1888_MOESM1_ESM.docx]

**Supplementary Material**

**Escitalopram promotes recovery from hand paresis in cortical sensorimotor stroke – a randomized, double-blind, placebo-controlled longitudinal study**

Vanessa Vallesi, Werner Krammer, Andrea Federspiel, John H. Missimer, Manuela Pastore-Wapp, Georg Kägi, Roland Wiest, Bruno J. Weder

**Table of content:**

1. Table ST1. Detailed inclusion and exclusion criteria

2. Table ST2. Correlations of behavioral data and age in normal volunteers tested (n = 28)

3. Table ST3. Weight of the seven dexterity hand parameters using PCA

4. Table ST4. Quality control of discrimination between verum and placebo groups using permutation testing

5. Table ST5. Lesion distribution in the patients (n = 21)

6. Table ST6. Estimated Marginal Means from the Generalized Estimating Equations Repeated-Measures Model

7. ST7. Pairwise Contrasts Between Placebo vs. Verum Subgroups at each Time Point from the Generalized Estimating Equations Repeated-Measures Model

8. Table ST8. Mean Differences in BOLD Activity Among Verum, Placebo, and Healthy Control Groups During Fixation vs. Observation

9. Table ST9. Mean Differences in BOLD Activity Among Verum, Placebo, and Healthy Control Groups During Fixation vs. Manipulation

10. SF1. Extension of the averaged lesions in the patients (n=21)

**Table ST1. Detailed inclusion and exclusion criteria**

**Inclusion Criteria**

Participants were eligible for inclusion if they had experienced a first-ever stroke with clinically significant contralesional hand plegia or paresis as the primary symptom. Neuroimaging evidence of involvement of the precentral and/or postcentral gyrus was required, as confirmed by diffusion-weighted imaging (DWI) and fluid-attenuated inversion recovery (FLAIR) sequences.

**Exclusion Criteria**

Participants were excluded if they had a history of psychiatric or neurological illness (e.g., brain tumor), were under 18 years of age, were pregnant, or were receiving any of the following medications: antidepressants; NMDA receptor agonists or antagonists; dopamine agonists or antagonists; levodopa; benzodiazepines; amphetamines; methylphenidate; foscarnet; ganciclovir; ritonavir; mianserin; chloroquine; mefloquine; imipenem; penicillin; ampicillin; cephalosporins; metronidazole; isoniazid; levofloxacin; cyclosporin; chlorambucil; vincristine; methotrexate; cytosine arabinoside; lithium; anticholinergics; systemic antihistamines; or systemic sympathomimetics. Additional exclusion criteria included any contraindication to magnetic resonance imaging (e.g., the presence of a pacemaker).

| **Table ST2. Correlations of behavioral data and age in Healthy Controls (n = 28)** | | | | | | | | | | | | | | | | | | | | | | | | | | | | | |  |  |  |  |
| --- | --- | --- | --- | --- | --- | --- | --- | --- | --- | --- | --- | --- | --- | --- | --- | --- | --- | --- | --- | --- | --- | --- | --- | --- | --- | --- | --- | --- | --- | --- | --- | --- | --- |
| Variables | | | | r | | r2 | | | intercept | | | t | | p (2-tailed) | | | df | slope | | | Lower 95% CI | | | | Upper 95% CI | | |  | | | |  |  |
|  |  |  |  |  |  |  |  |  |  |  |  |  |  |  |  |  |  |  |  |  |  |  |  |  |  |  |  |  |  |  |  |  |  |
| **Elementary sensori-motor parameters** | | | | | | | | | | | | | | | | | | | | | | | | | | | | | |  |  |  |  |
| PPT  vs age | | | | 0.53 | | | 0.28 | | | 1.12 | | 3.2 | | | 0.004 | 26 | | 0.047 | | | | 0.02 | | | | 0.08 | | | |  |  |  |  |
| Power  grip vs age | | | | -0.42 | | | 0.18 | | | 66.8 | | -2.38 | | | 0.025 | 26 | | -0.47 | | | | -0.88 | | | | -0.063 | | | |  |  |  |  |
| Precision grip vs age | | | | -0.19 | | | 0.04 | | | 11.1 | | **-1.0** | | | **0.32** | 26 | | -0.04 | | | | **-0.121** | | | | **0.04** | | | |  |  |  |  |
| **Dexterous hand parameters** | | | | | | | | | | | | | | | | | | | | | | | | | | | | | | | | | |
| TOR  vs age | | | -0.465 | | | | 0.22 | | | 31 | | -2.7 | | | 0.012 | | 26 | -0.04 | | -0.07 | | | | -0.01 | | | | |  | | | | |
| FG  vs age | | | | 0.085 | | | 0.007 | | | 0.98 | | **-0.4** | | | **0.664** | 26 | | -0.001 | | | | **-0.006** | | | | **0.004** | | | |  |  |  |  |
| JTT 1  vs age | | | -0.084 | | | 0.007 | | | 6.81 | | **-0.43** | | | **0.67** | 26 | | -0.014 | | | **-0.084** | | | | **0.055** | | |  | | | |  |  |  |
| JTT2  vs age | | | 0.56 | | | 0.31 | | | 0.55 | | 3.4 | | | 0.002 | 26 | | 0.1 | | | 0.04 | | | | 0.16 | | |  | | | |  |  |  |
| JTT 3  vs age | | | 0.53 | | | 0.28 | | | -1.13 | | 3.17 | | | 0.004 | 26 | | 0.11 | | | 0.04 | | | | 0.173 | | |  | | | |  |  |  |
| JTT 4  vs age | | | 0.77 | | | 0.6 | | | 0.5 | | 6.1 | | | <0.0001 | 26 | | -0.05 | | | 0.033 | | | | 0.066 | | |  | | | |  |  |  |
| JTT 5  vs age | | | 0.59 | | | 0.35 | | | 1.72 | | 3.72 | | | 0.001 | 26 | | 0.026 | | | 0.012 | | | | 0.041 | | |  | | | |  |  |  |

Abbreviations: df, degrees of freedom; CI, confidence interval; PPT, pressure perception threshold; JTT, Jebsen-Taylor Test; FG, ginger gaiting. In bold: the values indicate no dependence on age in the tested population (range 42 to 85 year

**ST3. Weight of the seven dexterity hand parameters using PCA**

In order to distinguish between the medicated patients and those receiving a placebo, seven behavioral measures acquired at each examination of the twenty-one patients were submitted to a sequence of PCAs constituting an Occam’s rasor. These measures included finger gating frequency (FG), TOR, and the five Jebsen-Taylor subtests: JTT1, JTT2, JTT3, JTT4 and JTT5. Before PCA, they were converted to z-scores using corresponding measures from the healthy control group such that more negative scores indicated increased impairment. Four of the fourteen patients were unable to complete a task at admission or later; they were excluded from the PCAs leaving nine patients receiving verum and eight placebo. With the MATLAB function *pca.m*, PCAs of the behavioral measures were computed. Each PCA yielded a number of components equal to the number of measures analyzed, percent of variance explained by a component, expression coefficients describing the degree to which a behavioral measure contributed to the component, and 17 patient scores describing the degree to which each patient contributes to the component. The principal components were ordered according to the percentage of variance explained; components contributing to a cumulative percentage of 80%, usually the first two, were admitted to further analysis. To be considered salient, the principal component must have exhibited significant discrimination, p<0.05, between the PC scores of the two patient subgroups. Further analysis of the patient score distribution estimated the discrimination threshold.

The application of Occam’s razor utilized the component expression coefficients. Behavioral measures contributing dominant expression coefficients were retained in the succeeding analysis. The PCA of the original seven measures showed the dominance of FG, TOR, JTT1 and JTT2 at each examination. Analyses of the PCAs including these four measures suggested that FG and JTT1 would be sufficient for discrimination.

In order to validate the most discriminating PCA, we performed permutation testing. The set of behavioral measures: FG and JTT1, were permuted separately for each subgroup, yielding 92 = 81 combinations of measures for the verum subgroup and 82=64 for the placebo subgroup. To avoid extreme combinations, only those were included in further analysis that yielded Mahalanobis distances - computed using the MATLAB function *mahal.m* as the Euclidean distances from the mean of the healthy controls in the two-dimensional space of FG and JTT1 scores - lying within the range of distances determined by the measures of the healthy controls: 71 combinations for verum and 61 for placebo patients. From each accepted permutation was computed a simulated patient score by projection of the expression coefficients. By applying the MATLAB function *perfcurve.m*, the combined distributions of simulated patient scores generated a receiver operating characteristic (ROC) curve that determined the optimal operating point, ie threshold, for discrimination and the rates of true and false positives. These could then be compared with the original patient score distribution.


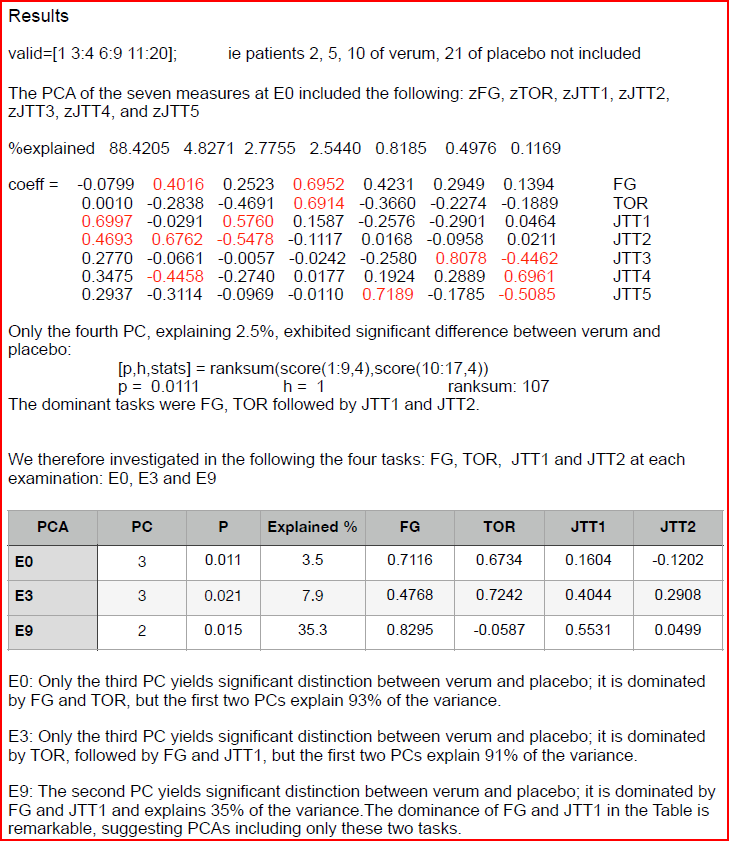


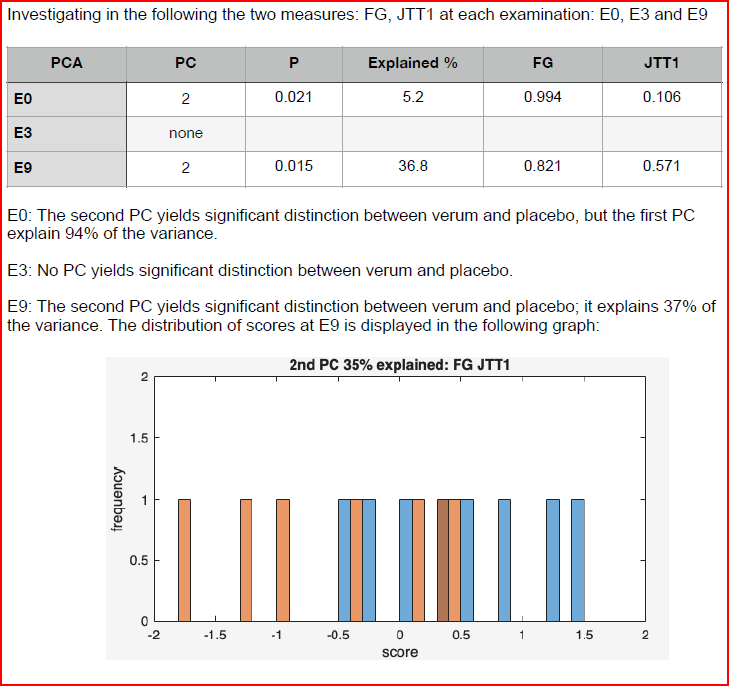


**
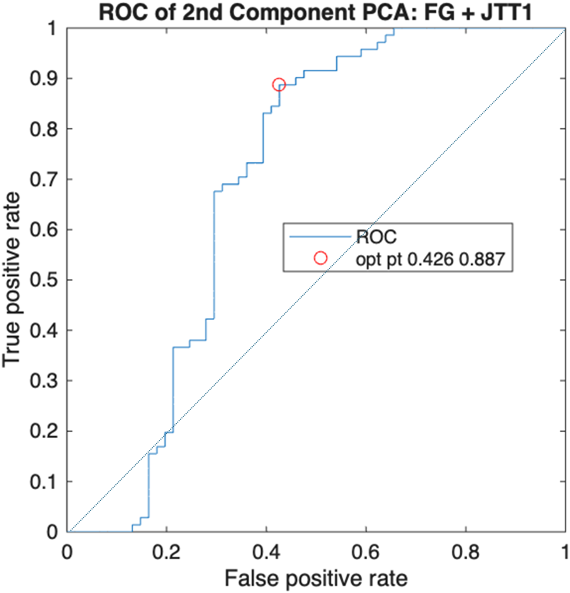
**

At the optimal point, corresponding to a true positive rate of 89% and false positive rate of 43%, the critical score in the above graph is: -1.1154, AUC 0.70

**ST4. Quality control of discrimination between verum and placebo groups using permutation testing**

FG and JTT1 For each set of measures, eg FG e0 or JTT1 e3, there are 12 verum and 9 placebo patient scores; these are ordered with the 12 verum scores first. Assigning the verum scores the value 1 and the placebo scores the value 0 yields a second ordered set. Permuted ordered sets result from partitioning a uniform distribution of 21 continuous random numbers between 0 and 1 into ones and zeros according to the ratio of verum patients to total number of patients: 12/21. This ratio assures that the most frequent number of ones in the distribution is 12. The scores of the original ordered set are then assigned values of 1 and 0 according to the permuted order. The difference of the medians of the scores assigned one and of those assigned zero are computed. The resultant distribution of median differences is compared to the median difference determined from the original ordered set of measures to determine the likelihood that the original scores could occur by chance. For the scores computed below, 60000 random distributions of ones and zeros were generated, of which about 10000 yielded the desired partition of 12 ones and 9 zeros.

**
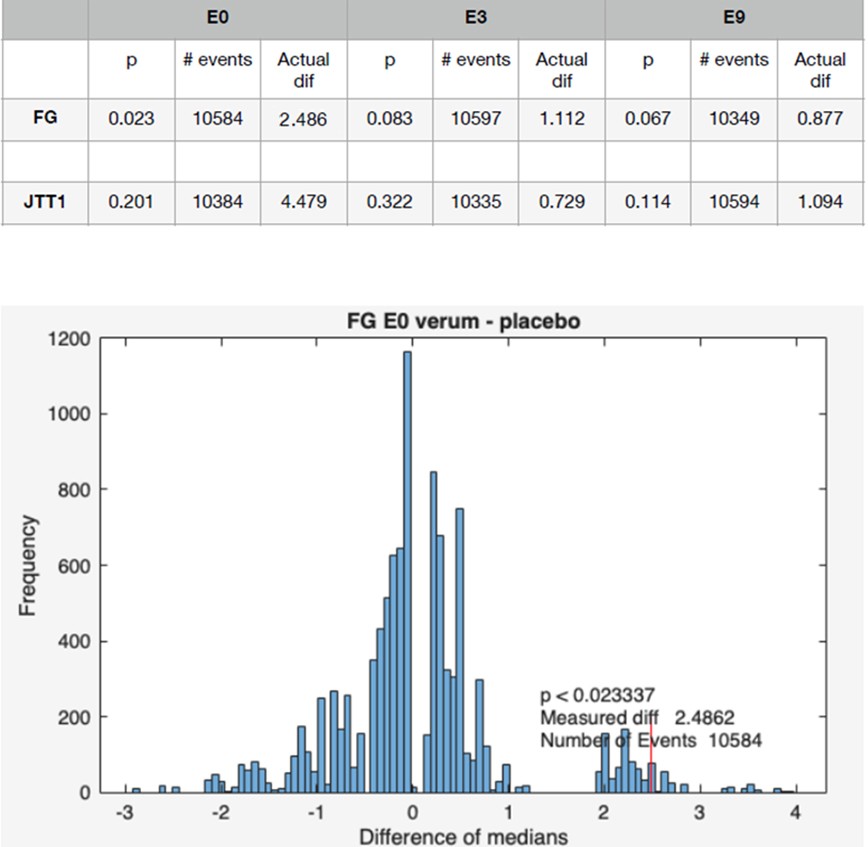
**

| **Table ST5. Lesion distribution in the patients (n = 21)** | | | | | | | | | | | | | | | | | | | |
| --- | --- | --- | --- | --- | --- | --- | --- | --- | --- | --- | --- | --- | --- | --- | --- | --- | --- | --- | --- |
|  | | | | | | | | | | | | | | | | | | | |
| **Patient ID** | | **Side** | **PMC** | | **4a** | **4p** | | **3a** | **3b** | | **1** | **2** | | **Underlying White Matter** | | | **Additional Cortical Areas** | | |
| 1 | | L | 6d1, 6d2+ | |  | + | | + |  | |  |  | | + | | | SPL-7A, IPS-hIp3 | | |
| 3 | | R |  | |  | + | | + | + | |  | + | | + | | | SPL-7PC, IPS-hIp1-2 | | |
| 4 | | L | 6d1+ | | + | + | | + | + | |  |  | | + | | |  | | |
| 5 | | R |  | |  | + | |  | + | | + | + | | + | | | SPL-5L,7PC, IPS-hIp1/3 | | |
| 6 | | L |  | | + | + | | + | + | | + | + | | ++ | | | SPL-5L, 7PC, IPL-PFm, IPL-Pga, PGp, IPS-hIp5-6/8, Visual-hOc4la/p | | |
| 8 | | R |  | | + | + | |  | + | | + | + | | + | | | IPS-hIp2 | | |
| 9 | | L |  | |  |  | |  | + | | + | + | | (+) | | | SPL-5L,7PC, IPS-hIp3, MFG-BA8, hOc4d | | |
| 10 | | L | 6d1-3+ | | + | + | | + | + | | + | + | | ++ | | | SPL-7A, IPL-PF, PF-t/cm/op, IPL-PGa, PGp, IPS-hIp1-3/5-8, hpO1, MFG-BA8-9, fOPC-Op8, OP1-2, Id6-7, Ig1/3, hOc4lp | | |
| 11 | | L |  | |  | + | | + | + | |  | + | | + | | | SPL-5L | | |
| 12 | | R | 6d1-3+ | | + | + | | + | + | | + | + | | + | | | SPL-7A/7P, IPS-hIp4-5/7-8, hOc4la/p, hOcV5/MT, hOc1-2/3d, frontal Pole, ITG | | |
| 13 | | R | 6d1-3+ | |  | + | | + | + | |  | + | | + | | | IPL-PFt | | |
| 14 | | R |  | |  |  | | + | + | |  | + | | + | | | SPL-5Ci, 7PC, hIp1-3 | | |
| 15 | | L |  | |  | + | | + | + | |  | + | | + | | |  | | |
| 16 | | L | 6d1-3+ | | + |  | | + | + | |  | + | | + | | | SPL-5L/7A/7P/7PC, IPL-PF, PFM, IPL-Pga, PGp, IPS-hIp1-5/7-8, hpO1, hOc2/3d/4d/4lp, MFG-BA8 | | |
| 17 | | L | 6d1-3+ | | + | + | |  |  | | + | + | | + | | | SPL-7A,7PC, IPS-hIp3, hOc2/3d, dlPFC* | | |
| 18 | | L | 6d1+ | | + | + | | + |  | |  |  | | + | | |  | | |
| 20 | | R | 6d1-3+ | | + | + | | + | + | |  |  | | (+) | | |  | | |
| 21 | | R | 6d1, 3+ | |  | + | | + | + | |  | + | | ++ | | | SPL-7PC, IPL-PFt, PFm, PGa, IPS-hIp1-3, dlPFC, MFG-BA8 | | |
| 22 | | L | 6d1, 3+ | |  | + | | + | + | | + | + | | + | | | SPL-7A,7PC, IPL-PFt, PFm, IPS-hIp2,6 | | |
| 23 | | L |  | |  | + | | + |  | |  | + | | (+) | | | SPL-5L, 7PC, IPS-hIp3 | | |
| 24 | | L | 6d1, 2+ | |  |  | |  |  | |  |  | | (+) | | |  | | |
|  | | | | | | | |  |  | |  |  | |  | | |  | | |
| Abbreviations: Cytoarchitectonically defined anatomical areas (Julich Cytoarchitectonic Atlas 3.1 (EBRAINS, Siibra): PMC: Premotor cortex (6d1-3); Motor cortex (4a, 4p, 3a, 3b, 1, 2); SPL: Superior parietal lobule (5L, 5Ci, 7PC, 7A, 7P); IPL: inferior parietal lobule (PF, PFt, PFcm, PFm, Pfop, Pga, PGp); IPS: Intraparietal sulcus (hIp1-8, hpO1); Visual cortex: (hOc2, 3d, 4d, 4la, 4lp, 5M, V5/MT); fOPC: Frontal opercular cortex (OP8); pOPC: Parietal Operculum (OP1, 2); Insula: (Id6, 7; Ig1, 3); dlPFC: Dorsolateral prefrontal cortex; MFG: Middle frontal gyrus; BA: Brodmann area; ITG: Inferior temporal gyrus; White matter lesion (+) minimally, + mildly and ++ moderately underlying the cortical layer   \| **Table ST6. Estimated Marginal Means from the Generalized Estimating Equations (GEE) Repeated-Measures Model** \| \| \| \| \| \| --- \| --- \| --- \| --- \| --- \| \| Group \| Timepoint \| EMM \| SE \| 95% CI \| \| Healthy controls \| Baseline(e0) \| 0.815 \| 0.156 \| [0.51, 1.12] \| \| Placebo-group \| Baseline(e0) \| -0.162 \| 0.732 \| [-1.60, 1.27] \| \| Verum-group \| Baseline(e0) \| -0.359 \| 1.01 \| [-2.34, 1.62] \| \| Placebo-group \| 3 Months (e3) \| 1.414 \| 0.37 \| [0.69, 2.14] \| \| Verum-group \| 3 Months (e3) \| 0.262 \| 0.313 \| [-0.35, 0.88] \| \| Placebo-group \| 9 Months (e9) \| 1.163 \| 0.416 \| [0.35, 1.98] \| \| Verum-group \| 9 Months (e9) \| 0.863 \| 0.338 \| [0.20, 1.53] \| \| Abbreviations: EEM, estimated marginal mean; SE, standard error; CI, confidence interval.  Estimated marginal means (EMMs) of global BOLD signal obtained from the GEE model; in patients, EMMs reflect model-adjusted values at each time point, and in healthy controls, the adjusted mean at baseline. Note the relatively low EMMs in the patient subgroups, the progressive normalization in the verum group, and the sharp increase to high values in the placebo group. \| \| \| \| \| | | | | | | | | | | | | | | | | | | | |
| \| **Table ST7. Pairwise Contrasts Between Placebo vs. Verum Subgroups at each Time Point from the Generalized Estimating Equations Repeated-Measures Model** \| \| \| \| \| \| --- \| --- \| --- \| --- \| --- \| \| Timepoint \| Estimate \| SE \| t-value \| p-value \| \| Baseline(e0) \| 0.197 \| 1.247 \| 0.158 \| 0.875 \| \| 3 Months (e3) \| 1.152 \| 0.485 \| 2.377 \| 0.018 \| \| 9 Months (e9) \| 0.3 \| 0.536 \| 0.56 \| 0.576 \| \| Abbreviations: SE, standard error.  Paired contrasts between placebo and verum subgroups at each time point from the repeated measures model with generalized estimating equations. Note the significant difference between the EEMs of both patient subgroups, indicating high global BOLD activity in the placebo group. \| \| \| \| \| | | | | | | | | | | | | | | | | | | | |
| **Table ST8. Mean Differences in BOLD Activity Among Verum, Placebo, and Healthy Control Groups During Fixation vs. Observation** | | | | | | | | | | | | | | | | | | | |
| **Baseline (e0)** | | | | **F-Value_(2, 41)_** | | | **P-Value_FWE_** | | | **MNI coordinates** | | | | | | **Healthy controls vs placebo-group** | | **Healthy controls vs verum-group** | **Placebo-group vs verum-group** |
|  |  |  |  |  |  |  |  |  |  | **X** | | | **Y** | | **Z** |  |  |  |  |
|  | R PCG, area 6v3, 6v2 | | | 22.71 | | | 0.001 | | | 54 | | | 5 | | 35 | -0.76^ns^ [-3.07, 1.54] | | -2.17*  [-4.19, -0.16] | -1.41^ns^  [-4.09, 1.27] |
| **3 Months (e3)** | | | |  | | |  | | |  | | |  | |  |  | |  |  |
|  | L inf, middle occ g, area hOc5 | | | 35.08 | | | < 0.001 | | | -40 | | | -75 | | -1 | -1.39^ns^  [-4.63, 1.84] | | -4.64**  [-7.64, -1.63] | -3.24^ns^  [-7.02, 0.53] |
| **9 Months (e9)** | | | |  | | |  | | |  | | |  | |  |  | |  |  |
|  | L inf occ g, area hOc5, hOc4la | | | 31.79 | | | < 0.001 | | | -42 | | | -76 | | 0 | -0.86^ns^  [-3.75, 2.03] | | -4.07***  [-6.59, -1.55] | -3.21^ns^  [-6.56, 0.15] |
|  | R inf occ g, area hOc4la | | | 32.70 | | | < 0.001 | | | 41 | | | -74 | | -1 | -2.39^ns^  [-6.11, 1.33] | | -5.78***  [-9.03, -2.54] | -3.39^ns^  [-7.71, 0.93] |
|  | R PoG, IPS, SPL, area 2, hIP3, 7PC | | | 23.46 | | | 0.001 | | | 31 | | | .36 | | 51 | -0.64^ns^ [-3.18, 1.89] | | -2.68*  [-4.89, -0.47] | -2.04^ns^  [-4.98, 0.91] |
|  | | | | | | | | | | | | |  | |  |  | |  |  |
| Abbreviations: *, p < 0.05; **, p < 0.01; ***, p < 0.001; ns, not significant; FWE, family-wise error; MNI, Montreal Neurological Institute; R, right; L, left; PCG, precentral gyrus; inf occ g, inferior occipital gyurs; PoG, postcentral gyrus; IPS, intraparietal sulcus; SPL, superior parietal lobule. | | | | | | | | | | | | | | | | | | | |
| Note: All p-values are family-wise error corrected for 0.05, and post-hoc tests are adjusted using Tukey HSD. The coordinates represent the center of gravity of the determined structure in each cluster.   \| **Table ST9. Mean Differences in BOLD Activity Among Verum, Placebo, and Healthy Control Groups During Fixation vs. Manipulation** \| \| \| \| \| \| \| \| \| \| \| \| \| \| \| \| \| \| \| \| \| \| \| \| \| --- \| --- \| --- \| --- \| --- \| --- \| --- \| --- \| --- \| --- \| --- \| --- \| --- \| --- \| --- \| --- \| --- \| --- \| --- \| --- \| --- \| --- \| --- \| --- \| \| **Baseline (e0)** \| \| \| **F-Value_(2, 41)_** \| \| **P-Value_FWE_** \| \| **MNI coordinates** \| \| \| \| \| \| \| \| \| **Healthy controls vs placebo-group** \| \| \| **Healthy controls vs verum-group** \| \| \| **Placebo-group vs verum-group** \| \| \| \| **X** \| \| \| **Y** \| \| \| **Z** \| \| \| \|  \| R PCG, area 6v1 \| \| 36.2 \| \| <0.001 \| \| 34 \| \| \| -13 \| \| \| 65 \| \| \| -5.51*  [-10.35, -0.68] \| \| \| -0.95^ns^  [-5.17, 3.27] \| \| \| 4.57^ns^  [-1.03, 10.16] \| \| \| \|  \| R IPL, area PFt, PFop \| \| 25.16 \| \| <0.001 \| \| 55 \| \| \| -24 \| \| \| 33 \| \| \| -2.97^ns^  [-7.02, 1.09] \| \| \| -3.95*  [-7.48, -0.41] \| \| \| -0.98^ns^  [-5.69, 3.73] \| \| \| \|  \| L post Putamen \| \| 26.35 \| \| <0.001 \| \| -31 \| \| \| -18 \| \| \| 6 \| \| \| -1.49*  [-2.93, -0.05] \| \| \| -0.07^ns^  [-1.20, 1.31] \| \| \| 1.54^ns^  [-0.13, 3.22] \| \| \| \|  \| R thalamus ve- la-po nc \| 27.52 \| \| <0.001 \| \| 15 \| \| -20 \| \| \| 10 \| \| \| -1.10*  [-2.13, -0.07] \| \| \| -0.67^ns^  [-1.57, 0.23] \| \| \| 1.43^ns^  [-0.77, 1.63] \| \| \| \|  \| L FOP, area OP6 \| \| 25.11 \| \| <0.001 \| \| -52 \| \| \| 6 \| \| \| 5 \| \| \| -0.53^ns^  [-2.01, 0.95] \| \| \| -1.53*  [-2.83, -0.24] \| \| \| -1.01^ns^  [-2.73, 0.72] \| \| \| \|  \| R PCG, area 6v3, 6v2 \| \| 24.52 \| \| <0.001 \| \| 59 \| \| \| 7 \| \| \| 31 \| \| \| **-2.61***  **[-5.20, -0.02]** \| \| \| **-2.85***  **[-5.12, -0.58]** \| \| \| -0.25^ns^  [-3.26, 2.77] \| \| \| \| **3 Months (e3)** \| \| \|  \| \|  \| \|  \| \| \|  \| \| \|  \| \| \|  \| \| \|  \| \| \|  \| \| \| \|  \| L SMA,area 6mp,  Cing. motor. area \| \| 73.38 \| \| <0.001 \| \| -8 \| \| \| -7 \| \| \| 64 \| \| \| 2.09*  [0.28, 3.90] \| \| \| 0.83^ns^  [-0.85, 2.51] \| \| \| -1.26^ns^  [-3.37, 0.85] \| \| \| \|  \| R FOP, area OP6,8; inf BA44 \| \| 25.46 \| \| <0.001 \| \| 51 \| \| \| 7 \| \| \| 2 \| \| \| -0.37^ns^  [-3.58, 2.85] \| \| \| -3.78*  [-6.77, -0.79] \| \| \| -3.41^ns^  [-7.15, 0.33] \| \| \| \|  \| L dlPFC, MFG4 \| \| 35.27 \| \| <0.001 \| \| -36 \| \| \| 41 \| \| \| 26 \| \| \| 2.45*  [0.36, 4.55] \| \| \| 0.46^ns^  [-1.48, 2.41] \| \| \| -1.99^ns^  [-4.44, 0.46] \| \| \| \|  \| L FOP, area 6, post inf BA44 \| \| 21.71 \| \| 0.001 \| \| -50 \| \| \| 6 \| \| \| 4 \| \| \| 1.88**  [0.62, 3.13] \| \| \| -0.09^ns^  [-1.26, 1.08] \| \| \| **-1.97****  **[-3.4, -0.50]** \| \| \| \|  \| R PCG, area 6v3 \| \| 29.65 \| \| <0.001 \| \| 57 \| \| \| 7 \| \| \| 30 \| \| \| 0.52^ns^  [-1.78, 2.82] \| \| \| -2.37*  [-4.51, -0.24] \| \| \| **-2.90***  **[-5.58, -0.21]** \| \| \| \|  \| L post putamen \| \| 25.01 \| \| <0.001 \| \| -30 \| \| \| -17 \| \| \| 6 \| \| \| 0.54^ns^  [-0.04, 1.11] \| \| \| -0.15^ns^  [-0.68, 0.38] \| \| \| **-0.69***  **[-1.36, -0.02]** \| \| \| \|  \| L anterior Insula Id6, Id7,Id8 \| \| 19.76 \| \| 0.003 \| \| -30 \| \| \| 19 \| \| \| 10 \| \| \| 1.59***  [0.73, 2.44] \| \| \| 0.16^ns^  [-0.63, 0.96] \| \| \| **-1.42****  **[-2.41, -0.43]** \| \| \| \| **9 Months (e9)** \| \| \|  \| \|  \| \|  \| \| \|  \| \| \|  \| \| \|  \| \| \|  \| \| \|  \| \| \| \|  \| R PCG, PoCG \| \| 37.62 \| \| <0.001 \| \| 38 \| \| \| -27 \| \| \| 51 \| \| \| -4.34**  [-7.74, -0.93] \| \| \| -1.38^ns^  [-4.35, 1.59] \| \| \| 2.95^ns^  [-1.00, 6.90] \| \| \| \|  \| L thalamus md and ant il nuclei \| \| 45.16 \| \| <0.001 \| \| -15 \| \| \| -23 \| \| \| 11 \| \| \| -0.80**  [-1.38, -0.22] \| \| \| 0.13^ns^  [-0.37, 0.64] \| \| \| **0.93****  **[0.26, 1.60]** \| \| \| \|  \| R dlPFC, area MFG4 \| \| 22.27 \| \| 0.001 \| \| 35 \| \| \| 38 \| \| \| 36 \| \| \| 0.16^ns^  [-2.10, 2.42] \| \| \| 2.65**  [0.68, 4.63] \| \| \| 2.49^ns^  [-0.14, 5.12] \| \| \| \|  \| R dlPFC, area  MFG4, MFG1 \| \| 26.84 \| \| <0.001 \| \| 40 \| \| \| 43 \| \| \| 25 \| \| \| 1.30*  [0.34, 2.26] \| \| \| 0.37^ns^  [-0.47, 1.21] \| \| \| -0.93^ns^  [-2.05, 0.18] \| \| \| \|  \| L visual cortex V1,area hOc1 \| \| 22.68 \| \| 0.001 \| \| -11 \| \| \| -66 \| \| \| 7 \| \| \| -0.66^ns^  [-2.12, 0.80] \| \| \| -1.28*  [-2.56, -0.01] \| \| \| -0.62^ns^  [-2.32, 1.08] \| \| \| \|  \| L Insula, Id6,Id7 \| \| 21.76 \| \| 0.002 \| \| -32 \| \| \| 18 \| \| \| 9 \| \| \| 1.09*  [0.00, 2.17] \| \| \| 0.58^ns^  [-0.36, 1.53] \| \| \| -0.51^ns^  [-1.77, 0.76] \| \| \| \|  \| \| \| \| \| \| \| \| \|  \| \| \|  \| \| \|  \| \| \|  \| \| \|  \| \| \| \| Abbreviations: *, p < 0.05; **, p < 0.01; ***, p < 0.001; ns, not significant; FWE, family-wise error; MNI, Montreal Neurological Institute; R, right; L, left; PCG, precentral gyrus; PoCG, postcentral gyrus; IPL, inferior parietal lobule; OP, operculum; SMA, supplementary motor area; MFG, middle frontal gyrus; BA, Brodmann area; PoG, postcentral gyrus; cing., cingulate; md, medial-dorsal. \| \| \| \| \| \| \| \| \| \| \| \| \| \| \| \| \| \| \| \| \| \| \| \| \| All p-values are family-wise error corrected at the 0.05 level, and post-hoc tests are adjusted using Tukey HSD. The aim was to investigate whether significant long-term differences emerge between the escitalopram and placebo subgroups at time points e3 and e9. These areas are indicated by bold p-values in the corresponding column, along with normalized BOLD activity in the verum subgroup at e3 and enhanced BOLD activity in the placebo subgroup at e9 compared to healthy volunteers, and are summarized in Table 4 of the manuscript. \| \| \| \| \| \| \| \| \| \| \| \| \| \| \| \| \| \| \| \| \| \| \| \|   **SF1. Extension of the averaged lesions in the patients (n=21)**  **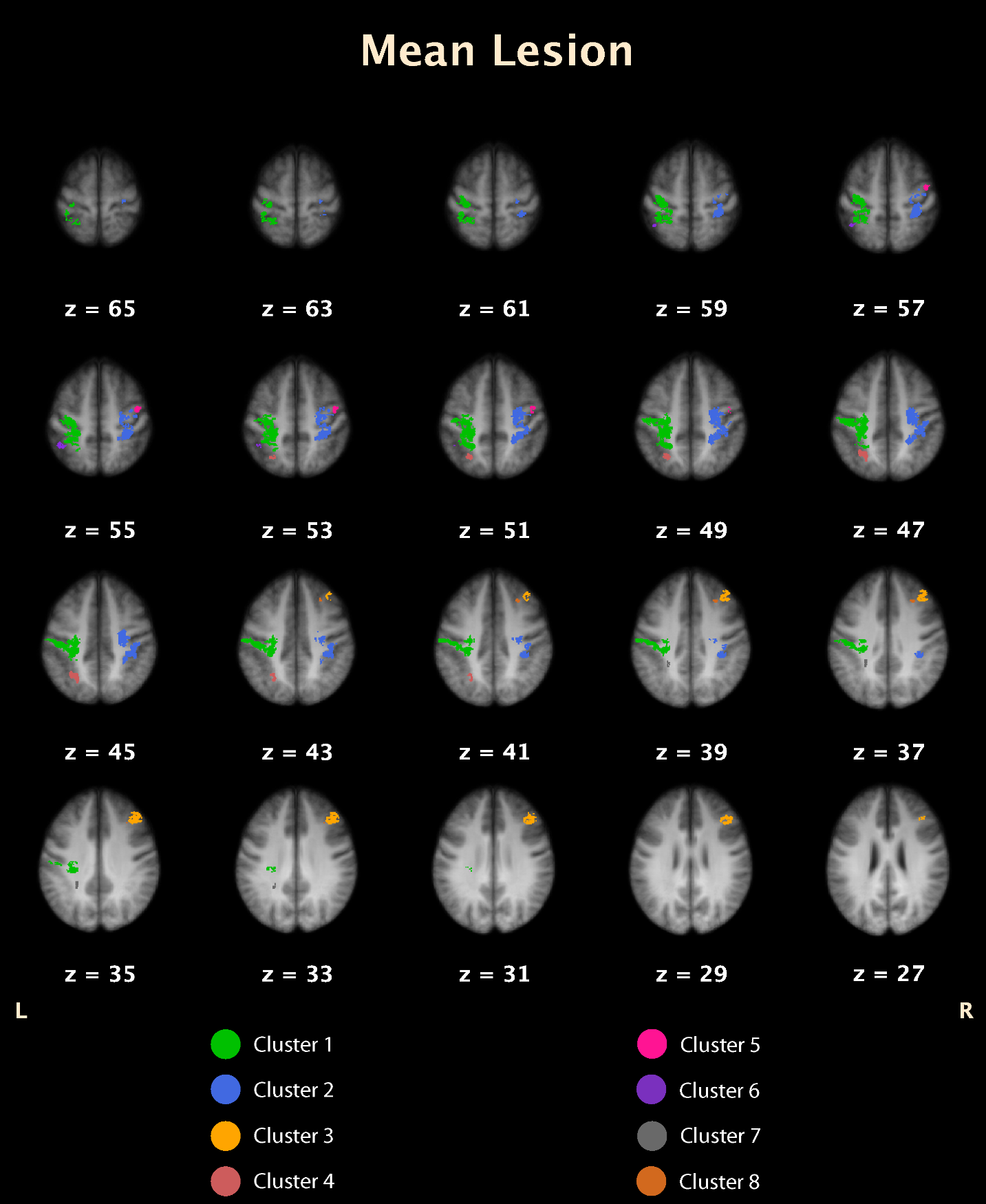**  The figure shows the averaged lesions demarcated by diffusion weighted imaging (DWI) and integrated into mean T1-weighted, transversal images from the healthy subjects using MRICro (Rorden and Brett, 2001). The delineated coloured ROIs mark lesion clusters which encompass 94 % of the entire lesion, four lesions each in the left and right hemisphere. The z-values relate to the cooordinate system of the MNI brain. Lesion size in the left hemisphere is 9.7 mm3, in the right hemisphere 7.3 mm3, no significant difference (t=0.47, p, = 0.65, two – tailed; F-test for the significance of the difference between the variances of the two samples F=1.93, p < 0.18). Lesion size in the verum group is 9.5 mm3, in the placebo group 7.6 mm3, no significant difference assuming unequal variances (t=0.37, p = 0.71, two – tailed; F-test for the significance of the difference between the variances of the two samples F=2.15, p < 0.14)).  Rorden C, Brett M. Stereotaxic display of brain lesions. Behav Neurol. 2000;12(4):191-200. doi: 10.1155/2000/421719. PMID: 11568431. | | | | | | | | | | | | | | | | | | | |
